# Methods for fine-mapping with chromatin and expression data

**DOI:** 10.1371/journal.pgen.1007240

**Published:** 2018-02-26

**Authors:** Megan Roytman, Gleb Kichaev, Alexander Gusev, Bogdan Pasaniuc

**Affiliations:** 1 Bioinformatics Interdepartmental Program, University of California Los Angeles, Los Angeles, California, United States of America; 2 Department of Medical Oncology, Dana Farber Cancer Institute, Boston, Massachusetts, United States of America; 3 Division of Genetics, Brigham and Women’s Hospital, Boston, Massachusetts, United States of America; 4 Department of Human Genetics, David Geffen School of Medicine, University of California, Los Angeles, Los Angeles, California, United States of America; 5 Department of Pathology and Laboratory Medicine, David Geffen School of Medicine, University of California, Los Angeles, Los Angeles, California, United States of America; University of Chicago Department of Medicine, UNITED STATES

## Abstract

Recent studies have identified thousands of regions in the genome associated with chromatin modifications, which may in turn be affecting gene expression. Existing works have used heuristic methods to investigate the relationships between genome, epigenome, and gene expression, but, to our knowledge, none have explicitly modeled the chain of causality whereby genetic variants impact chromatin, which impacts gene expression. In this work we introduce a new hierarchical fine-mapping framework that integrates information across all three levels of data to better identify the causal variant and chromatin mark that are concordantly influencing gene expression. In simulations we show that our method is more accurate than existing approaches at identifying the causal mark influencing expression. We analyze empirical genetic, chromatin, and gene expression data from 65 African-ancestry and 47 European-ancestry individuals and show that many of the paths prioritized by our method are consistent with the proposed causal model and often lie in likely functional regions.

## Author summary

Genome-wide association studies (GWAS) have revealed that the majority of variants associated with complex disease lie in noncoding regulatory sequences. More recent studies have identified thousands of quantitative trait loci (QTLs) associated with chromatin modifications, which in turn are associated with changes in gene regulation. Thus, one proposed mechanism by which genetic variants act on trait is through chromatin, which may in turn have downstream effects on transcription. In this work, we propose a method that assumes a causal path from genetic variation to chromatin to expression and integrates information across all three levels of data in order to identify the causal variant and chromatin mark that are likely influencing gene expression. We demonstrate in simulations that our probabilistic approach produces well-calibrated posterior probabilities and outperforms existing methods with respect to SNP-, mark-, and overall path-mapping.

## Introduction

Discerning the genetic and molecular basis of complex traits is a fundamental problem in biology. Genome-wide association studies have revealed that the majority of variants associated with disease lie in noncoding regulatory sequences [1, 2]. Identifying the target genes of these variants and the mechanisms through which they act remains an open problem [3]. Recent efforts to systematically characterize how genetic variation impacts more granular molecular phenotypes have yielded thousands of single nucleotide polymorphisms (SNPs) that associate with local and distal histone modifications—termed histone quantitative trait loci (hQTLs) [4–7]. Furthermore, recent studies have identified many expression quantitative trait loci (eQTLs) that co-localize with hQTLs, implying there may exist a shared genetic influence on epigenetic traits and gene expression [8–11]. Therefore, one proposed mechanism by which regulatory variants may affect gene expression and thereby impact traits is through changes in chromatin state [10]. However, this putative chain of causality whereby the effects of SNPs on expression are mediated by chromatin modifications has yet to be established. This is further compounded by the complex space of plausible causal directions connecting transcription factor binding, DNA methylation, chromatin variation, and gene expression. Since laboratory experiments are very costly, there is a need for statistical methods that can accurately prioritize the causal SNP and chromatin mark within an implicated region under a plausible causal model. However, even if the causal direction is given, pinpointing the exact SNP and mark within a genomic region is very challenging due to the confounding effects of linkage disequilibrium (LD) among SNPs and correlations among marks [5, 6, 10, 12–14].

Methods to investigate the relationships between the genome, the epigenome, and expression have largely focused on quantifying the overlap between hQTLs and eQTLs [10, 14, 15]. Previous studies have sought to identify hQTLs by selecting the SNP with the strongest p-value for association to a local chromatin mark and to local gene expression [10, 14, 15]. Moreover, various methods exist for the fine-mapping of SNPs that may be concurrently affecting two traits, including eCAVIAR [16] and Coloc [17]. Although these methods can be applied to jointly analyze SNP, chromatin, and expression data, they do not model the causal path whereby SNPs impact expression through chromatin alteration.

Here we propose a fine-mapping framework, *pathfinder*, that explicitly models the hierarchical relationships between genome, chromatin, and gene expression to predict both the causal SNP and the causal mark within a gene region that are influencing expression of a given gene. Our framework assumes a causal model where a SNP impacts a chromatin which in turn alters gene expression. In our framework we refer to a “causal” SNP as any SNP that disrupts inter-individual variation of chromatin state either through a direct biological mechanism (e.g., chromatin accessibility) or indirectly through an unobserved biological mechanism. Similarly, we refer to a “causal” chromatin mark as either a mark that biologically alters expression or that tags an underlying epigenetic regulatory mechanism of expression. Our framework takes as input the strength of association (as quantified through the standard Z-scores) between all SNP/mark pairs and all marks to expression as measured in a given set of individuals. To explicitly account for the correlation structure among SNPs and marks, we use a Matrix-variate Normal distribution to model all Z-scores jointly. By construction, this allows our probabilistic model to assign posterior probabilities for each SNP, mark, and path (where paths include all possible SNP-mark combinations) to be causal in the region. A key advantage of our approach is that it produces well-calibrated posterior probabilities for causality. Thus, *pathfinder* can be used to prioritize variants and marks for validation experiments.

In simulations we compare against several existing methods, demonstrating that *pathfinder* outperforms alternative approaches with respect to both accuracy and calibration. This is largely because our comparators do not take into account mark-expression associations. In some cases, these additional associations may help distinguish between two potentially causal paths that have comparable evidence for causality. For example, in cases where a SNP is associated with expression of a local gene and is also associated with two local chromatin marks, knowledge of the impact of each mark on gene expression may help distinguish between two possible paths for causality. Finally, we analyze genotype, chromatin and expression data from 65 African-ancestry and 47 European-ancestry individuals. We show that the top causal SNPs proposed by *pathfinder* tend to lie in more functional regions and disturb more regulatory motifs than expected by chance. We also present evidence that most of the top paths reported by *pathfinder* demonstrate consistency with our proposed sequential model, thus strengthening the case for our method’s applicability to empirical biological data.

## Results

### Overview of hierarchical fine-mapping with genetic, chromatin, and gene expression data

Here we introduce a hierarchical statistical method for fine-mapping of causal SNPs and chromatin marks (e.g., histone modifications) that may be concordantly influencing gene expression within a genomic region. We build upon previous insights that a vector of Z-scores is well-described by a Multivariate Normal (MVN) distribution parameterized by LD [13, 18, 19] to model association statistics between chromatin marks and gene expression. We analyze all chromatin peaks across four mark types (DHS, H3K4me1, H3K4me3, and H3K27ac) jointly in the same framework; we refer to a “mark” as a chromatin peak at a particular location, and “mark types” as DHS, H3K4me1, H3K4me3, and H3K27ac. To simultaneously take into account both SNP LD and the correlations between chromatin marks, we use the Matrix-variate Normal distribution to jointly model association statistics between all SNPs and marks within a region. Our method takes as input SNP-mark and mark-expression associations within a region centered around a particular gene, as well as correlations among all SNPs (LD) and correlations among all considered marks. *Pathfinder* enumerates over all possible causal paths, considering one causal SNP and one causal mark for each path, and outputs a posterior probability for each path to be causal, which can subsequently be used to prioritize SNPs and marks for validation. We compute marginal probabilities for individual SNPs (or marks) to be causal by summing the posterior probabilities over all paths that contain the SNP (or mark). For simplicity, in this work we refer to a “causal” mark as a mark that either causally drives inter-individual variation of gene expression or is correlated to an underlying causal mechanism (e.g. transcription factor binding), though it may not be biologically causal for expression.

**Fig 1:**
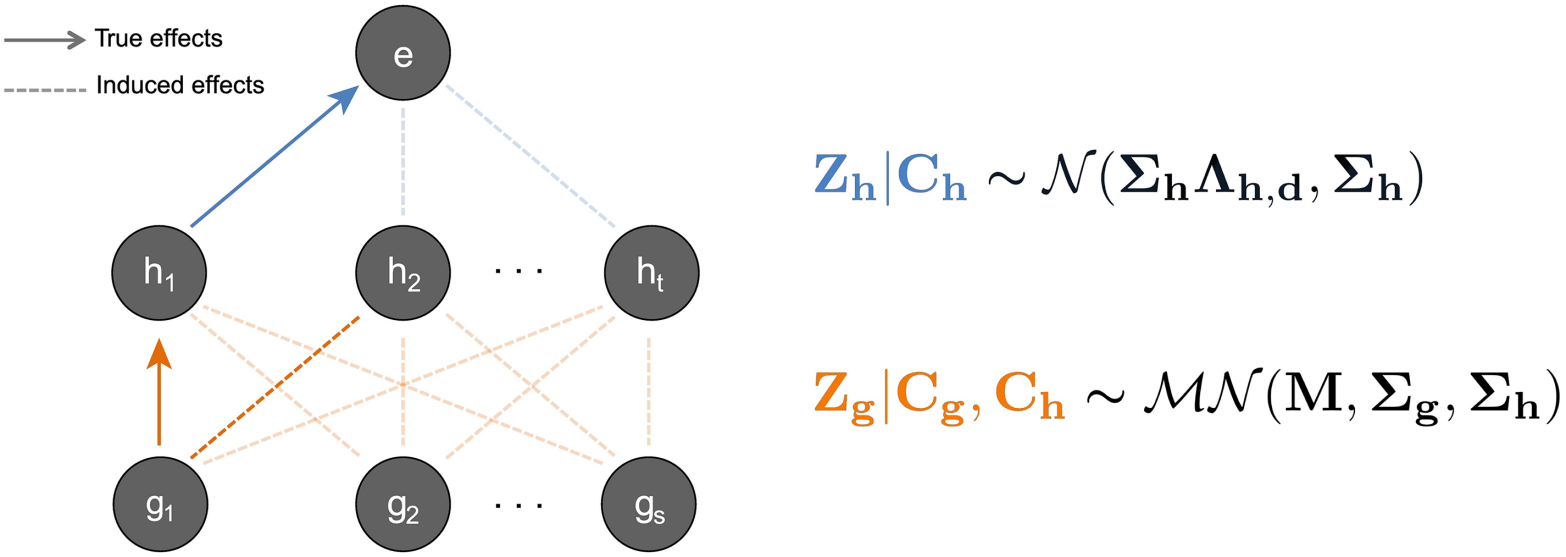
Schematic of hierarchical model whereby SNPs affect histone marks, which in turn affect gene expression. We illustrate a scenario where SNP *g*_1_ and mark *h*_1_ are causal. All other induced correlations, such as the effect of *g*_1_ on *h*_2_, are an effect of LD and/or correlations among marks. To the right we show our mathematical model for this hierarchical framework. On the top level, we model mark-expression associations with a Multivariate Normal (MVN) distribution. On the bottom, we jointly model all associations between all SNPs and marks with a Matrix Variate Normal distribution (see Methods).

The advantage of our method over existing approaches is that it integrates mark-expression associations which may help distinguish between two paths with otherwise comparable evidence for causality. We illustrate a scenario in Fig 1. Consider a genetic region where SNP *g*_1_ has a strong association with two local marks *h*_1_ and *h*_2_, as well as a significant association with gene expression. Using only SNP-mark and SNP-expression effects, we are unable to discern whether SNP *g*_1_ influences expression through mark *h*_1_ or *h*_2_. However, if we consider mark-expression effects, we see that mark *h*_1_ has a strong association with gene expression where mark *h*_2_ does not. This additional information helps support the hypothesis that there is a causal path from SNP *g*_1_ to mark *h*_1_ to gene expression.

### *Pathfinder* improves fine-mapping performance

We used simulations to compare *pathfinder*’s performance against alternative methods with respect to SNP-, mark-, and path-finding efficiency as well as the calibration of its posterior probabilities. We generated genetic, chromatin, and gene expression data for 10,000 50kb regions, each centered around a single gene, over 100 individuals, using SNP LD and mark correlations derived from 65 Yoruban (YRI) individuals (see Methods). We define a “mark” as an individual peak location for any mark type in the dataset (DHS, H3M4me1, H3K4me3, or H3K27ac). For each gene, we randomly assigned a single causal pathway from one SNP to one mark to gene expression. We then ran our methods on all regions individually and assessed their ability to correctly prioritize the true causal path in each region (Methods).

We compare against an independent fine-mapping approach (whereby we fine-map SNP-mark associations and mark-expression associations independently and take the product of the resulting probabilities to produce posterior probabilities for paths), a Bayesian network analysis [20], a naive ranking (where we rank SNP-expression and mark-expression associations to prioritize SNPs and marks within a region; for path-finding, we rank the product of these two), a formal colocalization method [17], and finally, against overlaps between eQTLs and hQTLs within a region centered around a gene of interest (see Methods). Unlike the first four approaches, the overlap methods do not produce rankings, but yield candidate sets of causal SNPs, marks, and paths. For this reason, we present these results in a separate analysis using an alternative metric for comparison.

**Fig 2:**
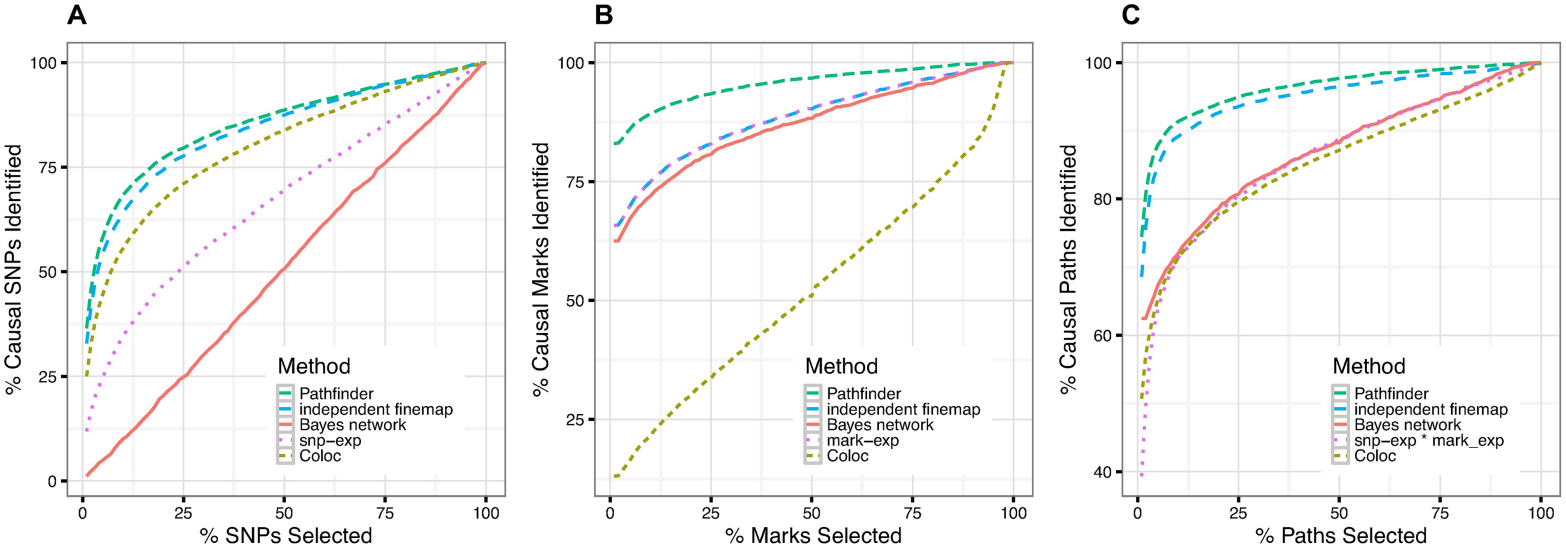
Comparison of our method against four potential competitors—Independent fine-mapping, a simple ranking of associations, Coloc, and Bayesian network analysis. We measure performance as the number of simulated causal SNPs, marks, and paths that each method is able to recapture, while varying the number of SNPs, marks, or paths considered.

We find that *pathfinder* has consistently better performance than the other ranking approaches with respect to all three features—SNP-, mark-, and path-mapping within a region (Fig 2). For example, association ranking, Coloc, Bayesian network analysis, and independent fine-mapping accumulate 55%, 62%, 47%, and 13% of the top paths on average in order to recapture 90% of the causal paths, whereas our method only requires 8% of the top paths. Note that SNP-expression association ranking is equivalent to running a basic eQTL analysis, which does not take into account chromatin data, in order to identify causal SNPs. A similar improvement in accuracy was observed for the size of the credible sets, defined as the number of SNPs required to capture a given percentage of the causal variants (S1 Table).

**Fig 3:**
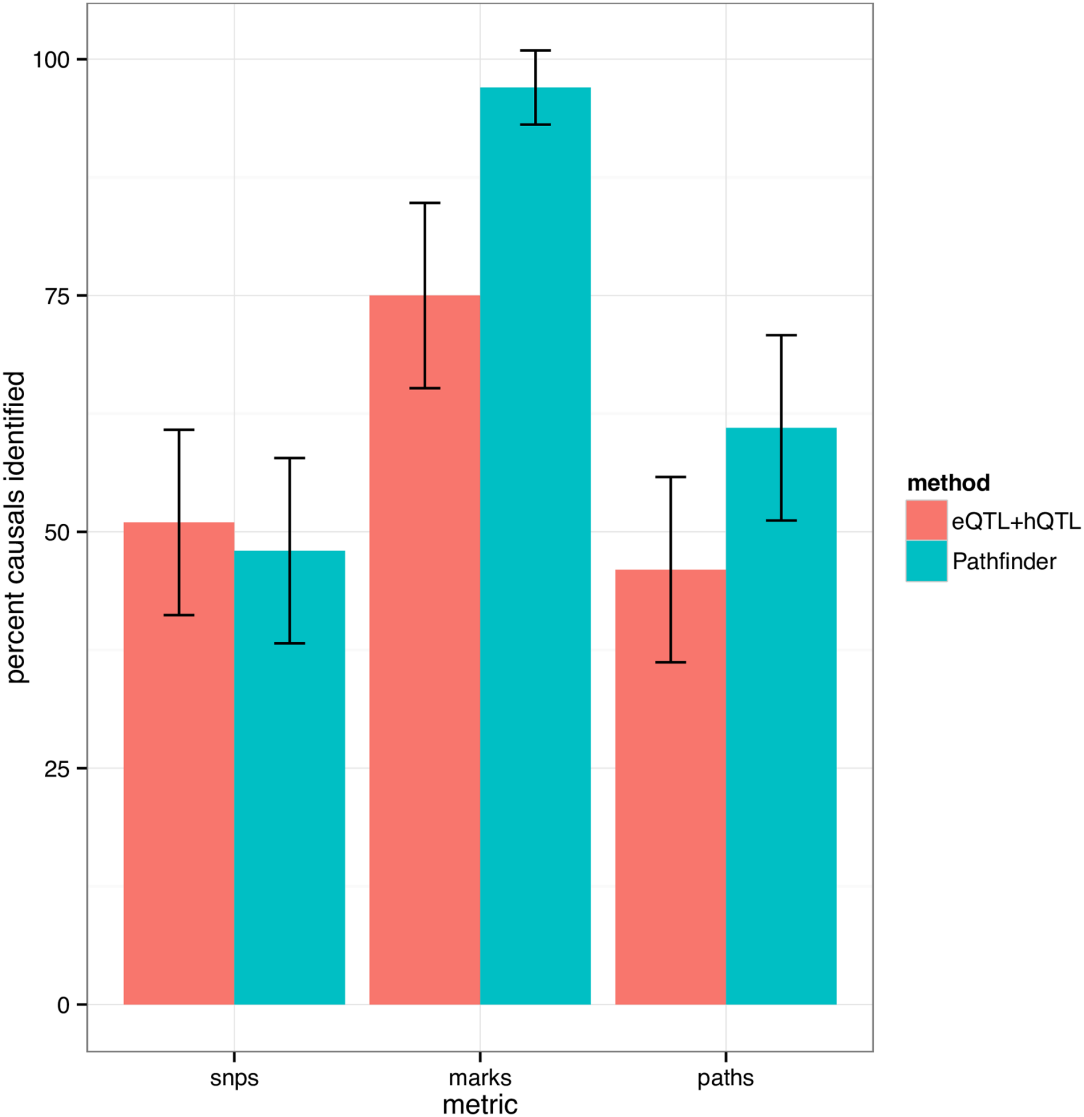
Comparison of our method to standard eQTL + hQTL overlap analyses. In overlap analyses, only the top SNP for association to each histone mark and gene expression is considered. We demonstrate significant gains in our method with respect to mark-finding accuracy, where SNP-mapping performance is comparable between the two methods.

Next, we evaluated *pathfinder*’s performance compared against standard analyses that investigate overlaps between hQTLs and eQTLs within a genomic region. In such experiments, the variant with the strongest association to each local chromatin mark is selected, as well as the variant with the strongest association to local gene expression. In addition, marks are filtered to ensure a 10% FDR (see Methods). This produces a set of candidate marks, as well as one candidate SNP per mark, and one SNP deemed causal for gene expression in the region. Implicitly, the overlap of these variants suggests a set of candidate SNPs, marks, and paths for the region. For the same set sizes, *pathfinder* identifies 96% of the causal marks versus 74% in the standard overlap approach (Fig 3). SNP-finding accuracy is comparable between the two methods.

**Fig 4:**
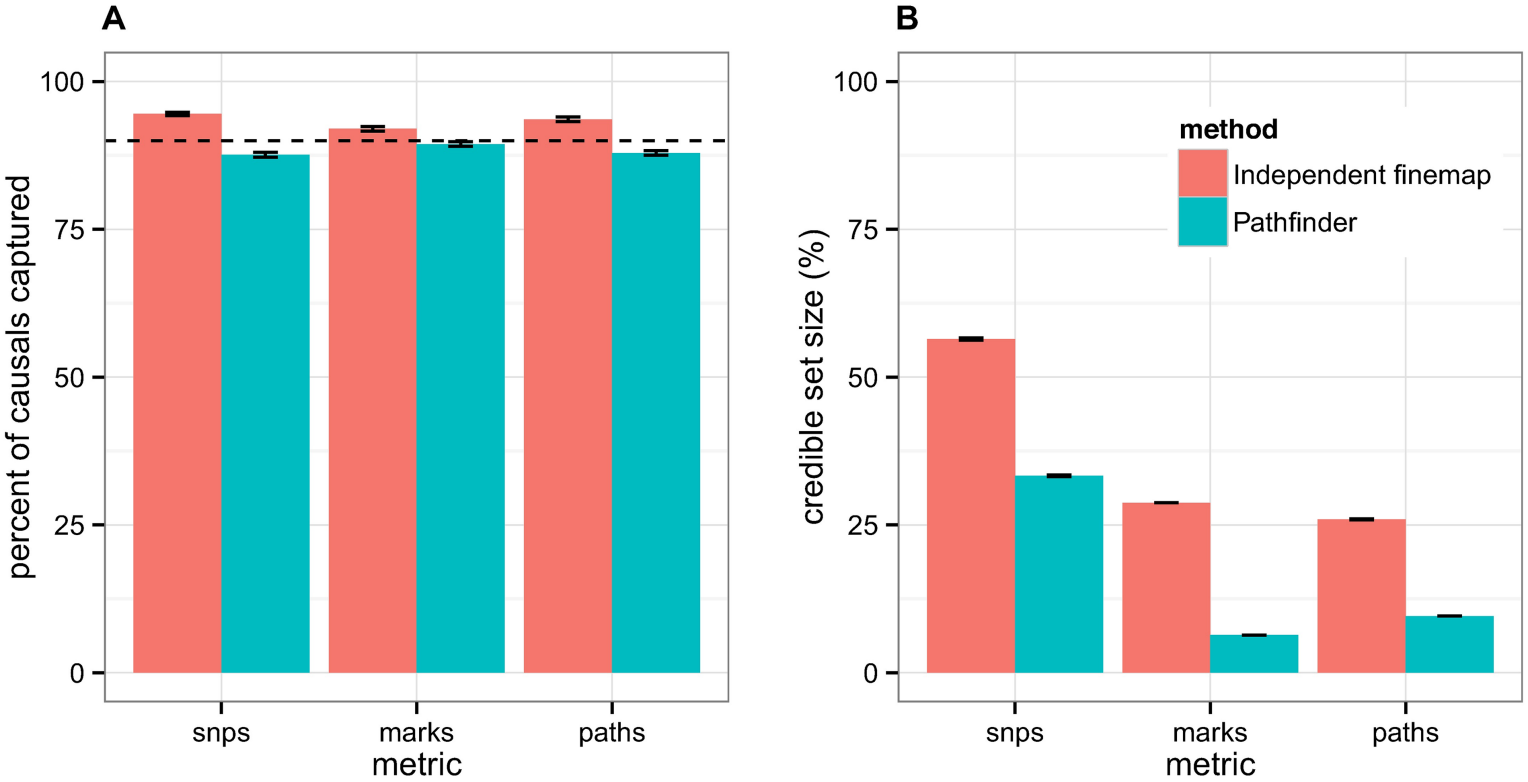
90% credible sets for SNP-, mark-, and path-mapping. We compare *pathfinder* to the technique of independently fine-mapping the two levels of data, with respect to (A) the calibration of their credible sets and (B) the size of their credible sets. In (A), we compare the proportion of causal variants that were captured in the 90% credible sets using *pathfinder* vs. independent fine-mapping against the expected proportion (represented by the dotted line). In (B), we display the corresponding sizes of these credible sets.

We next assessed the calibration of the posterior probabilities for causality output by *pathfinder*. Our method has slightly deflated credible sets for SNP- and path-finding, but well-calibrated credible sets for mark-finding (Fig 4). In contrast, the independent fine-mapping approach has consistently inflated credible sets—that is, it captures more causal paths than expected, but also has drastically larger credible set sizes. For example, when accumulating 90% of the posterior probabilities over all regions, *pathfinder* captures 88% of the true causal paths within the top 380 candidate paths, whereas independent fine-mapping captures 94% of the causal paths within the top 1026 candidate paths. Similar outcomes were attained for the 50% and 99% credible sets (S1 Fig). Overall, *pathfinder*’s credible sets are less biased and narrower than those obtained through the independent fine-mapping approach.

**Fig 5:**
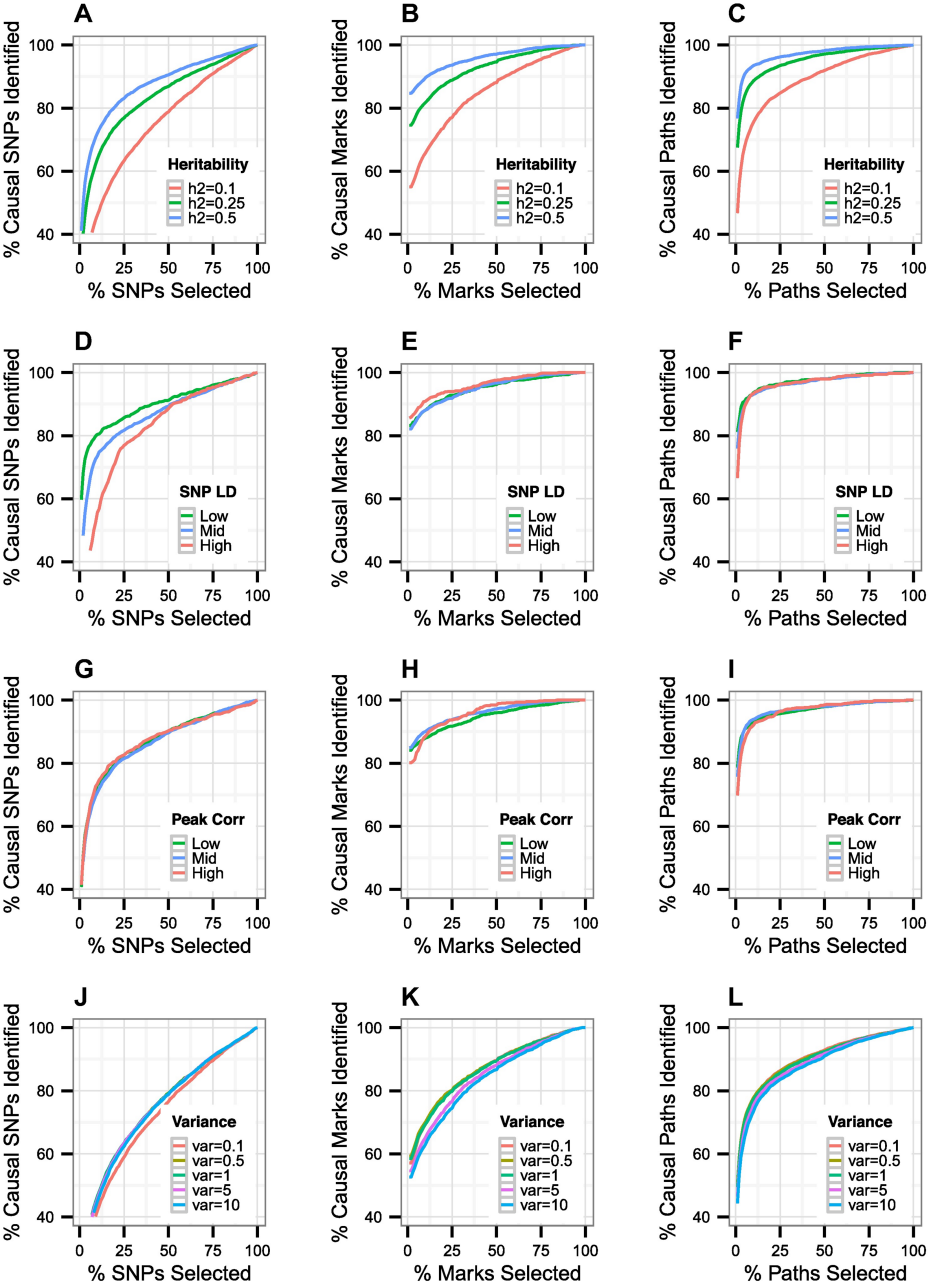
Performance of our method as we vary levels of variance explained, SNP LD, mark correlations, and the prior variance parameter. (A-C) We simultaneously vary the variance explained by SNP and mark from 0.1 to 0.5 per region. (D-I) We stratified based on mean SNP/mark correlations at the causal SNP/mark. (J-L) We show that *pathfinder* is not sensitive to variations in our prior variance parameter.

Finally, we investigated the effects of simulation and method parameters on *pathfinder*’s accuracy. Firstly, we varied the causal SNP and mark effect sizes such that the variance explained of mark and gene expression ranged from 0.1 to 0.5. As anticipated, increased heritability leads to better performance (See Fig 5A–5C). Secondly, in order to assess the impact of SNP LD and mark correlations on SNP- and mark-finding performance, we stratified our existing simulations based on the mean correlation of the causal SNP or mark to all other SNPs or marks (See Fig 5D–5I). We grouped our simulations into three categories: low, medium, and high correlations. As anticipated, SNP-finding performance decreases slightly as SNP LD increases. Notably, mark-finding performance is actually improved at higher SNP LD. This is due to the redundancy in information about SNP-mark associations at the causal mark when these effects are exhibited across multiple correlated SNPs. SNP- and mark-finding performance, however, do not seem to be significantly affected by mark correlations in our simulations—at least not at the level of variation exhibited in our data. In addition to stratifying our existing simulations by LD, we also assessed the impact of using European rather than African LD in the same regions, as European LD is known to be more extensive. Here we retained the YRI mark and expression data in order to isolate the effect of SNP correlations. The credible set sizes computed from the CEU dataset do not substantially differ from those obtained in YRI (S2 Table). This result demonstrates that the more extensive LD observed in European individuals will not significantly affect *pathfinder*’s performance. Thirdly, we evaluated the effect of the prior variance tuning parameter on fine-mapping performance (See Fig 5J–5L). The prior variance is an estimate of the variance explained by the causal SNP and mark in the region, as we do not know a priori what the causal effect sizes are. We show that the optimal range for the prior variance parameters is between 5 and 10, in simulations with a variance explained of 0.25 on both levels. Overall, performance does not seem to change drastically in response to variations in the prior variance, even significantly outside of this optimal range.

### Violations of the model

**Fig 6:**
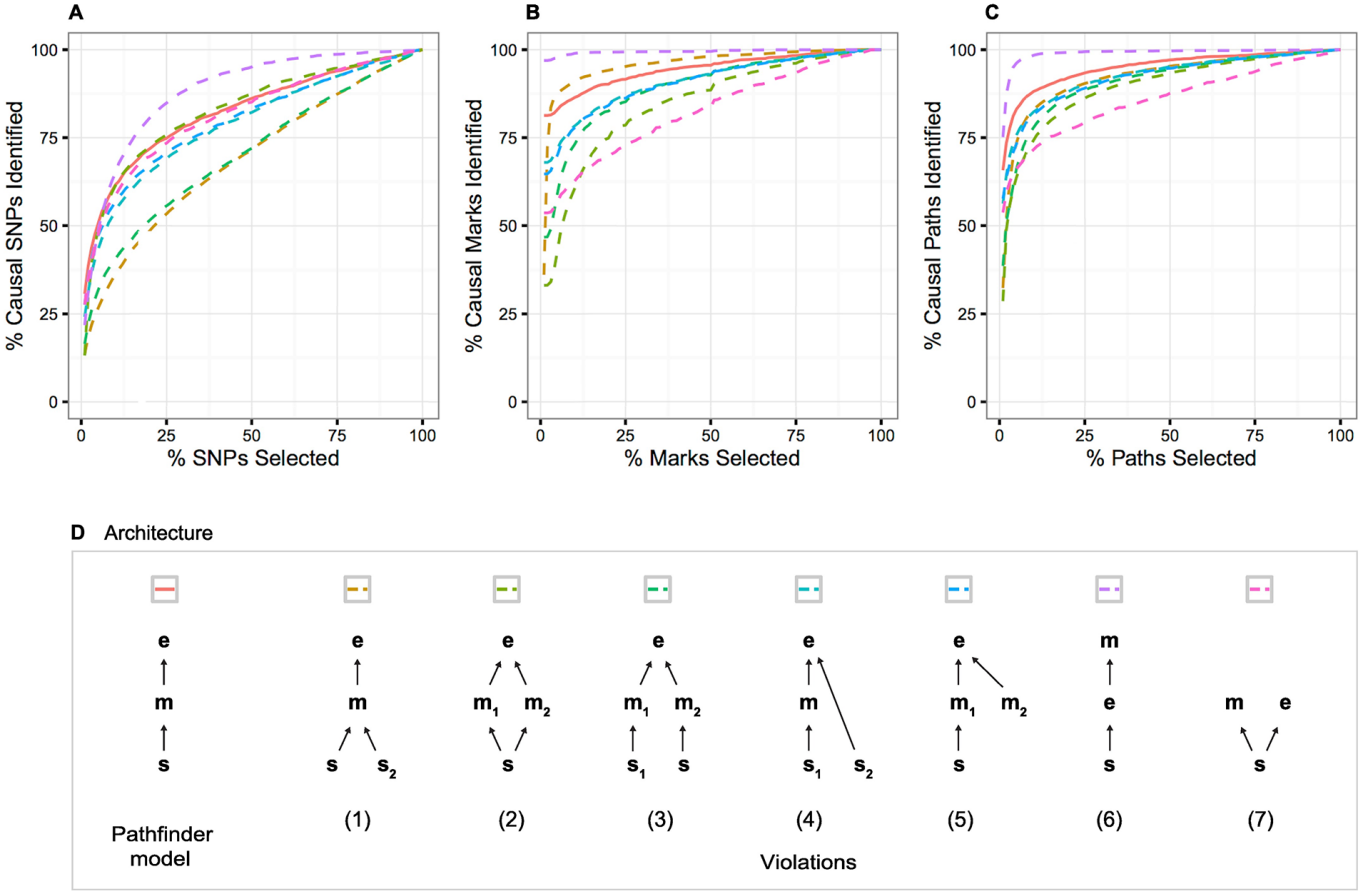
Performance of our method under violations of the causal model. (A-C) *pathfinder*’s SNP-, mark-, and path-mapping accuracy for standard simulations compared with seven model violations. (D) The model violations include the following scenarios: (1) multiple causal SNPs impact a single causal mark, which affects gene expression, (2) a single SNP impacts multiple causal marks, which both affect gene expression, (3) two SNPs affect two marks (respectively), which both impact gene expression, (4) a single causal SNP impacts a single causal mark that affects gene expression, with an additional SNP also impacting gene expression directly, (5) a single causal SNP impacts a single causal mark that affects gene expression, with an additional mark also impacting gene expression, (6) a single causal SNP affects gene expression directly, which in turn affects a single mark, and (7) a single causal SNP has independent effects on a single mark and gene expression.

Our hierarchical model makes several key assumptions that may sometimes be violated in empirical data. Firstly, *pathfinder* assumes that a single causal SNP and a single causal mark are driving the associations within a region, where in reality there may exist multiple true causal SNPs or marks [13, 19]. Secondly, *pathfinder* assumes that SNP effects on gene expression are mediated by a chromatin mark, which may not be the case in real data. We therefore assessed the performance of our method when these two assumptions are violated in various ways, diagrammed in Fig 6.

First, we investigate violations 1–3, which include multiple causal pathways throughout the region. Path-mapping accuracy, measured by the proportion of causal paths identified, is reduced in all three scenarios (Fig 6). Note that the number of causals identified does not necessarily decrease, but rather the proportion, as there are more causal paths in each region. SNP- and mark-finding accuracy under these violations are also compromised, but with two notable exceptions. In the multi-causal-SNP scenario, mark-finding accuracy increased in comparison with the single-SNP simulations; for example, only 8% of marks were selected (versus 18% in the single causal simulations) to capture 90% of the causal marks. In the multi-causal-mark scenario, SNP-finding accuracy increased. Intuitively, this is due to the redundancy in the signal that is captured by the Matrix-variate Normal distribution.

We next investigate violations 4–5, in which an additional SNP or mark influences gene expression directly. We observe in these two scenarios that performance is reduced for SNP-, mark-, and path-finding, but not drastically. For example, in order to capture 90% of the causal paths, *pathfinder* must select on average 25% and 28% of paths under violations 4 and 5, respectively (compared with 15% under standard simulations). Because anti-correlated marks (e.g. activating and repressing marks) often tend to act in the same region, we also assess *pathfinder*’s behavior specifically when two marks have opposite effects on expression. As expected, *pathfinder*’s performance does not decline in the presence of anti-correlated peaks (S2 Fig).

Finally, we discuss *pathfinder*’s performance under violations where the causal order is modified (violations 6–7). Under violation 6, where a single causal SNP affects gene expression directly, which in turn affects a single mark, *pathfinder* actually captures a higher proportion of the affected marks and overall paths. For example, in order to capture 90% of the causal paths, *pathfinder* must select on average only 3% of the top-ranked paths (compared with 15% under standard simulations). In violation 7, where the SNP has independent effects on the mark and the gene expression, we show that *pathfinder*’s accuracy in finding the causal mark and path is significantly reduced. Note that in this case, the “path” is not truly a path but a SNP/mark pair, as effects of the SNP on mark and gene expression are independent. Our power in distinguishing between these two models depends on the prior variance explained parameter. Under violation 7, the variance explained in gene expression by the causal mark is much smaller than expected, thus reducing our confidence in the true causal configuration. We conclude that under the SNP→expression→mark violation, *pathfinder* will identify causal paths very confidently even if they do not follow the assumed SNP→mark→expression model. Therefore a high posterior probability for a path may not be sufficient evidence for causality. On the other hand, when SNP effects on mark and expression are independent, *pathfinder* is less likely to produce false positives. For these reasons, we recommend a pre- or post-filtering step to retain only those regions that show some prior evidence for the SNP→mark→expression model using a conditional analysis or partial correlation approach (Methods).

For completeness, we also assess existing methods under these simulations (S3 Fig). Most notably, the simple association-ranking approach shows a distinct improvement under violations 6 and 7, in which SNPs have a direct effect on gene expression. This is expected as *pathfinder* assumes the causal effect to be mediated by chromatin. A similar improvement can be observed for Coloc under violation 7, in which the SNP affects both chromatin and gene expression directly.

### Empirical data analyses

We evaluated the behavior of our hierarchical fine-mapping method when applied to empirical data. We performed these analyses on data from 65 YRI individuals whose genotypes were obtained through 1000 Genomes, and whose PEER-corrected H3K4me1, H3K4me3, H3K27ac, DHS, and RNA expression levels in lymphoblastoid cell lines (LCLs) were obtained from [10]. In each region, we analyzed all four mark types jointly (H3K4me1, H3K4me3, H3K27ac, and DHS) by including all peaks spanning the region for each mark type. Each peak of each mark type was therefore treated as a single chromatin mark. We filtered the 14,669 regions using a two-step regression analysis to yield 1,317 regions that showed evidence for the sequential model of SNPs affecting histone marks which in turn affect gene expression (see Methods). *pathfinder*’s runtime scales approximately as *s*^3^*t*^3^, where *s* and *t* are the number of SNPs and marks within a region, respectively. On average, each 50kb region contained 160 SNPs and 25 marks. Most runs were completed in under a few minutes. The most dense region contained 331 SNPs and 66 marks and took approximately 21 minutes (S4 Fig).

In Table 1, we report the average 50%, 90%, and 99% credible set sizes produced when running *pathfinder* on real data. We compare against basic eQTL mapping, where we fine-map SNPs to gene expression ignoring chromatin data. We show that the credible set sizes are significantly narrower when running *pathfinder* with all three levels of data, consistent with our findings in simulations. For example, eQTL mapping requires an average of 45.3 SNPs in order to capture 90% of the posterior probability for SNP causality, whereas *pathfinder* only requires 28.4 SNPs. If we define a gene to be fine-mapped if 99% of the posterior probability mass for SNP causality is contained within the top 10 SNPs or fewer, then standard eQTL mapping fine-maps 46 of the genes in our data, whereas *pathfinder* fine-maps 73 of the genes. Notably, *pathfinder* also requires only 1.8 marks on average in order to capture 90% of the posterior probability for causal marks. In 82% of the regions where the top two marks capture more than 90% of the posterior probability, these two marks are two distinct peaks of the same mark type.

**Table 1:** 50%, 90%, and 99% credible sets for SNP-, mark-, and path-mapping for real data analysis. We compare *pathfinder* to basic eQTL mapping, with respect to the size of their credible sets, averaged across all regions. Standard errors are included next to each measurement.

method	50% credible set			90% credible set			99% credible set		
	SNPs	Marks	Paths	SNPs	Marks	Paths	SNPs	Marks	Paths
*pathfinder*	4.9 (0.2)	1.0 (0.0)	7.4 (0.3)	28.4 (1.1)	1.8 (0.1)	158.4 (6.0)	64.2 (2.4)	6.3 (0.2)	765.5 (29.0)
eQTL mapping	8.1 (0.3)	-	-	45.3 (1.7)	-	-	92.9 (3.6)	-	-

The mean variance explained observed in the top path chosen by *pathfinder*, across all conforming regions, were 0.38 (s.e. 0.01) for the SNP-mark effect and 0.20 (s.e. 0.01) for the mark-expression effect (S5 Fig). These effects are reasonably consistent with the 25% variance explained we used in simulations at each level (see Simulations). The correlation between the SNP-mark and mark-expression effect size magnitudes in the top selected paths across all regions was 0.03 (p = 0.400). That is, the strength of the SNP-mark effect did not seem to correlate with the strength of the mark-expression effect. We assessed the relative impacts of each type of histone mark by computing the proportion of probability mass assigned to each mark type in aggregate over all regions (S3 Table). H3K4me3 is the most informative mark type in this data, capturing 31% of the total probability mass despite being the least prevalent of all four mark types, constituting only 13% of all marks.

We also report the size of *pathfinder*’s credible sets when applied to empirical CEU data rather than YRI in Table 2. These two datasets are not directly comparable, as the types of epigenetic marks and their quantities differ substantially. Nonetheless, we demonstrate that *pathfinder*’s performance on the CEU dataset does not drastically diverge from its behavior in YRI. Data pre-processing strategies such as PCA and PEER correction may substantially impact the number of mark-expression correlations that are retained [21]. We find that credible set sizes for PEER-corrected data are narrower, giving a slight but significant improvement in performance (S4 Table).

**Table 2:** 50%, 90%, and 99% credible sets for SNP-, mark-, and path-mapping for simulations using empirical YRI and CEU data. We compare *pathfinder’s* performance when using SNP LD from YRI vs from CEU, with respect to the size of its credible sets, averaged across all regions. Standard errors are included next to each measurement.

method	50% credible set			90% credible set			99% credible set		
	SNPs	Marks	Paths	SNPs	Marks	Paths	SNPs	Marks	Paths
YRI	4.9 (0.2)	1.0 (0.0)	7.4 (0.3)	28.4 (1.1)	1.8 (0.1)	158.4 (6.0)	64.2 (2.4)	6.3 (0.2)	765.5 (29.0)
CEU	7.4 (0.3)	1.0 (0.0)	10.6 (0.4)	30.1 (1.1)	1.3 (0.0)	90.1 (3.3)	59.5 (2.2)	2.8 (0.1)	269.6 (9.8)

As our pre-filtering step was designed to preserve only regions in which SNP effects on gene expression are mediated by chromatin, we expected a large majority of the analyzed regions to show evidence for this mechanism. To confirm this, we investigated whether the top paths prioritized by our method demonstrate consistency with this causal model. We defined a set of top paths as those which were ranked first in a region and whose posterior probabilities for causality were assigned by *pathfinder* to be greater than 0.1. This resulted in 480 total top paths. Out of 480 top paths, only 12 had a significant (*p* < 0.05/480) partial correlation between SNP and gene expression after controlling for chromatin. However, 193 paths had a significant partial correlation between SNP and chromatin after controlling for gene expression. This finding suggests that the top paths are more consistent with the SNP→mark→expression model than with a SNP→expression→mark model.

**Fig 7:**
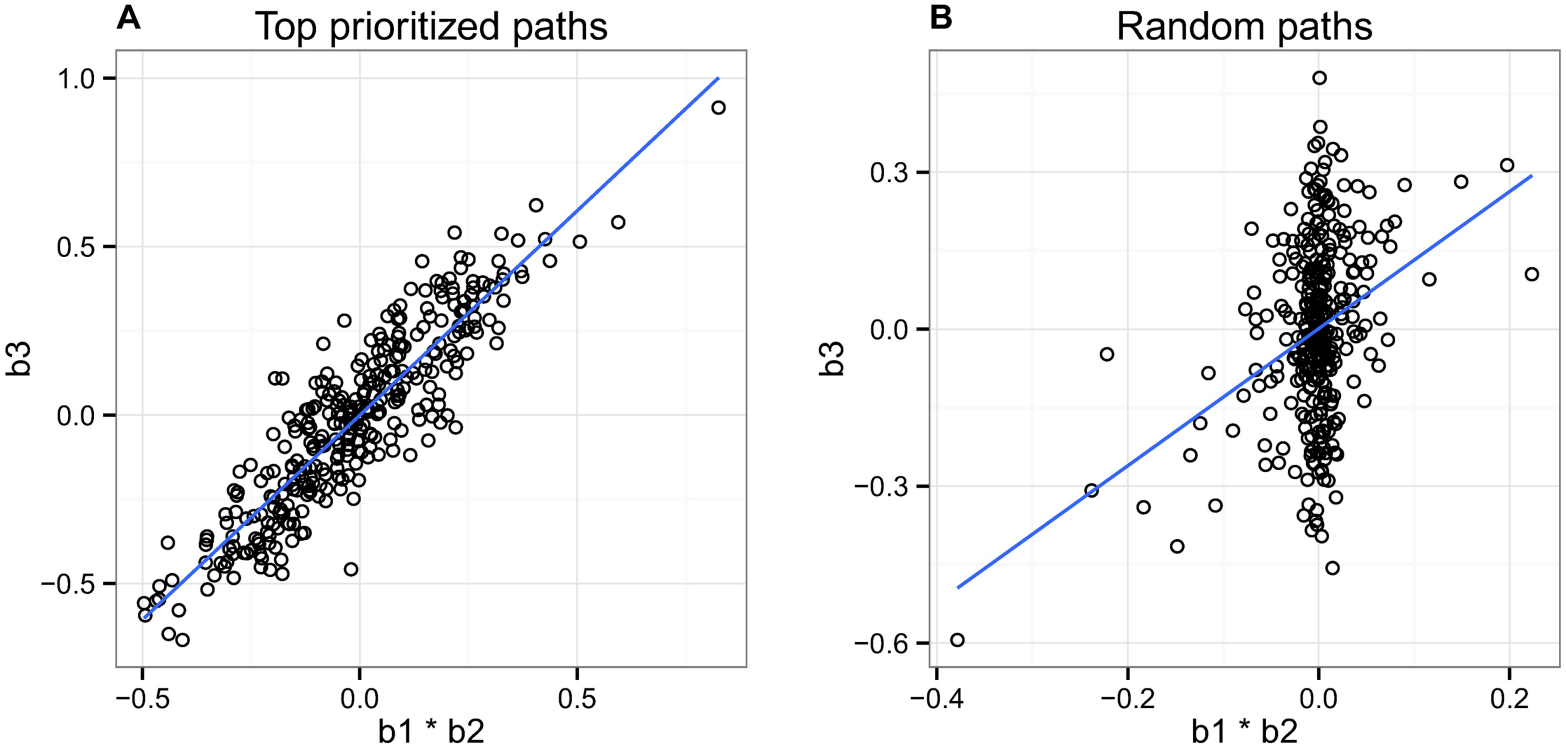
Relationship between the product of the SNP-mark and mark-expression effect sizes against the overall SNP-expression effect size. (A) We observe a high correlation (r = 0.91) between these effect size vectors, indicating that our method is identifying many pathways that are likely to be following our causal model. Here we included only the top paths whose posterior probabilities for causality were assigned to be greater than 0.1. (B) We show that a significant correlation does not exist for randomly chosen paths.

Next we examined the relationship between the product of the effect sizes between SNP-mark and mark-expression against the overall SNP-expression association (Fig 7). We expect this relationship to be correlative; if truly mediated by the mark in question, the overall SNP-expression effect size should be proportional to the product of the two contributing effect sizes. Note that we weight our correlation by the reported posterior probability for each path, such that the paths we have more confidence in will contribute more to this metric. We find a high correlation (r = 0.91) between these effect size vectors for our top paths, as compared with a correlation of r = 0.36 when running the same analysis on random paths within each region. This result indicates that *pathfinder* is identifying many pathways that are likely to be following its causal model.

**Fig 8:**
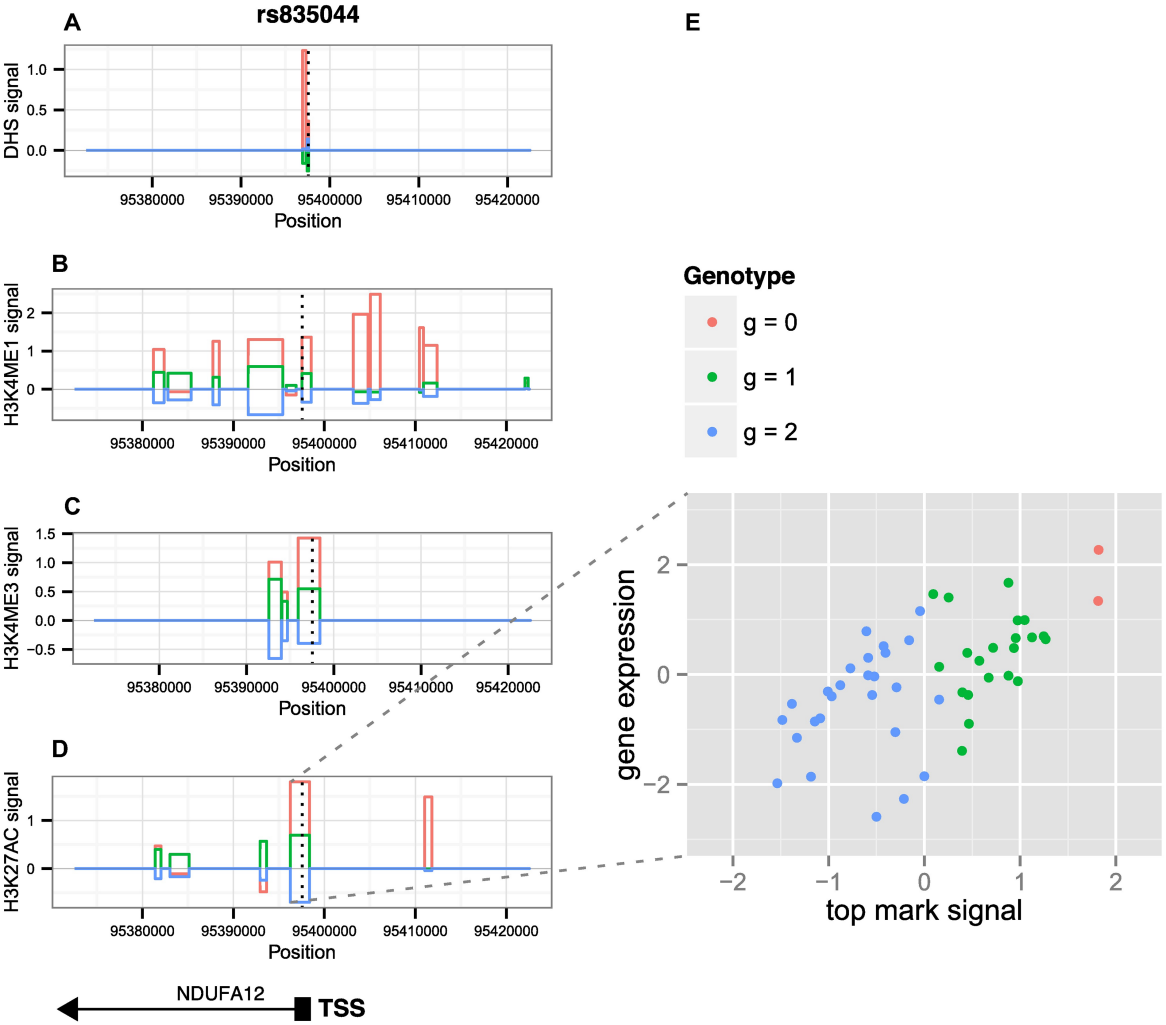
Genomic context of top path reported by *pathfinder* in real data. (A-D) Mark signals for DHS, H3K4me1, H3K4me3, H3K27ac in a 4kb region centered around the NDUFA12 TSS, stratified by genotype. The implicated SNP, signified by the vertical dotted line, lies 6bp downstream of the gene TSS, and falls within an H3K27ac peak, which is also the top mark reported by *pathfinder*. The posterior probability for causality for this peak was greater than 0.999. (E) Relationship between the H3K27ac peak signal and gene expression, stratified by genotype.

In Table 3, we list the top ten paths prioritized by *pathfinder* across all real data regions. Most SNPs implicated in these paths are known to alter several regulatory motifs and often lie in an enhancer region or a promoter region of the genes whose expression they affect. 59% (s.e. 2%) of the SNPs implicated in the top paths fall into active ChromHMM states (1–7) in LCLs, including active TSS, flanking active TSS, transcription at gene 5’ and 3’, strong transcription, weak transcription, genic enhancers, and enhancers. Only 47% (s.e. 2%) of random paths fall into these active states (p = 0.001834). Moreover, on average, SNPs in the top paths disturbed 5.35 (s.e. 0.26) regulatory motifs, whereas random SNPs chosen at the same regions only disturbed 4.40 (s.e. 0.20) motifs on average (p < 0.001). We did not, however, observe a similar change in transcription factor binding affinity at these motifs (*δ* = 5.26 vs *δ* = 5.27, (p = 0.511)). As an example, in Fig 8A–8D, we display the genomic context for the top region reported by *pathfinder*, including average mark signals for DHS, H3K4me1, H3K4me3, and H3K27ac, stratified by genotype, in a 4kb region centered around the TSS of the NDUFA12 gene. The implicated SNP lies within the NDUFA12 TSS. Fig 8E plots the gene expression signal against that of the top mark, stratified by genotype. In S6 Fig, we show associations for the top region reported by *pathfinder*, spanning a 50kb region centered around the NDUFA12 TSS.

**Table 3:** Top causal paths produced by real data analysis. For each path, we report the chromosome, the RSID of the implicated SNP, the implicated mark type, the posterior probability we assigned to this path, three Z-scores (SNP to mark association, mark to expression association, SNP to expression association), the GENCODE gene around which this region was centered, the ChromImpute [24] annotation for the SNP, and the number of regulatory motifs altered by the SNP, as designated by HaploReg [25].

chr	rsid	mark type	posterior	SNP-mark Z	mark-exp Z	SNP-exp Z	gene	chromatin state	motifs altered
12	rs835044	H3K27ac	> 0.99	-13.05	4.97	-4.65	NDUFA12	1TssA	5
1	esv3587154	H3K4me1	> 0.99	-18.13	17.40	-14.97	GSTM1	15Quies	-
19	rs385895	H3K4me1	> 0.99	12.60	2.41	1.50	CLC	7Enh	3
15	rs8025332	H3K4me1	> 0.99	-12.07	2.11	-2.35	CELF6	15Quies	1
5	rs1217817	H3K4me1	> 0.99	-14.59	5.58	-4.52	MAP1B	7Enh	4
1	rs7417106	DHS	> 0.99	-8.62	-0.16	-0.54	C1orf170	4Tx	22
1	rs111900551	H3K4me3	> 0.99	-8.82	2.26	-2.95	CLCNKA	15Quies	18
3	rs57339700	H3K4me1	> 0.99	-9.66	2.37	-2.29	CAND2	14ReprPCWk	5
6	rs9349050	H3K4me3	> 0.99	-12.47	10.80	-8.19	MDGA1	11BivFlnk	2
3	rs6763025	H3K4me1	> 0.99	10.59	-2.21	-2.18	PRSS50	7Enh	4

**Fig 9:**
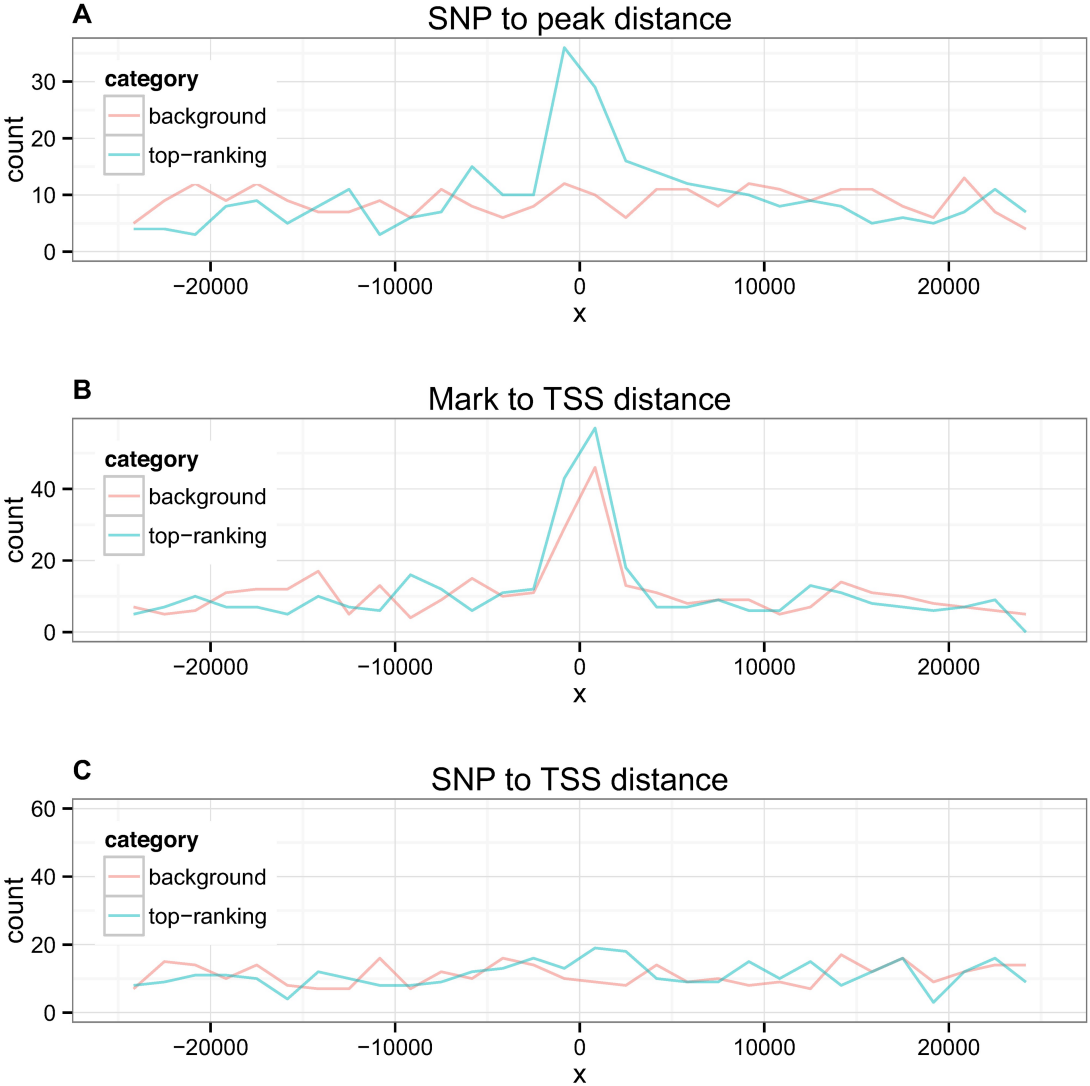
Spatial relationships between SNP, mark, and TSS in top paths reported by *pathfinder* vs random paths. (A) Distances from SNP to mark (B) Distances from mark to TSS (C) Distances from SNP to TSS.

Next we examined the spatial relationships between the SNP, mark, and TSS implicated in the top paths reported by *pathfinder* (Fig 9). SNP to mark and mark to TSS distances were significantly lower in our selected paths compared with randomly chosen paths at the same regions. The average distance from SNP to mark in *pathfinder*’s top paths was approximately 11.7kb, compared to 15.3kb in randomly chosen paths (p < 0.001). The average distance from mark to TSS in selected paths was approximately 8.6kb, compared to 9.7kb in randomly chosen paths (p = 0.026). SNP to TSS distances were not significantly different in top versus random paths (p = 0.108), with top SNPs lying on average 11.7kb away from the TSS and random SNPs lying 12.4kb away. 5% of top SNPs lied within 2kb of the TSS while 15% lied within 2kb of the corresponding peak. 23% of peaks in the top paths lied within 2kb of the gene TSS. S7 Fig displays all three distances where top paths are stratified by mark type.

To further validate the top paths chosen by *pathfinder*, we determined the extent to which SNPs in these paths overlap with eQTLs that have been identified in LCLs using the larger scale Geuvadis data set [22]. 21% of the top paths contained SNPs that were also identified as eQTLs from the Geuvadis data set. In comparison, when randomly choosing paths at the same regions, only 11% overlapped with eQTLs (p < 0.001). Simply choosing the SNP with the highest association with gene expression in each region (equivalent to standard eQTL-mapping) resulted in an overlap of 24% with existing eQTLs. These results contradict the improvement in accuracy demonstrated in simulations when using *pathfinder*. We suspect this discrepancy is due either to imperfect locus ascertainment (i.e., a number of loci may include SNPs that directly affect gene expression rather than indirectly through chromatin) or the fact that the Geuvadis eQTLs were also selected using standard fine-mapping approaches and we may thus expect a stronger agreement between the two resulting eQTL sets.

We also investigated the extent to which *pathfinder*’s top SNPs overlap with eQTLs that have been experimentally validated through differential expression in an LCL dataset [23]. Here, we define the set of validated eQTLs to be those whose p-values for differential expression passed a threshold of 0.01. We find that 2.2% (or 13) of *pathfinder*’s top SNPs overlap with this validated set, where choosing the SNP with the highest association with gene expression in each region resulted in an overlap of 2.3% (also 13 SNPs).

Finally, we investigated whether any of the top paths reported by *pathfinder* could be found within GWAS hit regions for various autoimmune diseases, as our data were collected from LCLs. These autoimmune diseases included Celiac disease, Crohn’s disease, PBC (Primary Biliary Cirrhosis), SLE (Systemic Lupus Erythematosus), MS (Multiple Sclerosis), RA (Rheumatoid Arthritis), IBD (Irritable Bowel Disease), and UC (Ulcerative Colitis). We restricted to GWAS hits with variants associated to the trait with *p* < 5 × 10^−8^. We found that 19 of our 480 top paths were contained in a GWAS-implicated region. In Table 4, we report the paths that localized within autoimmune GWAS regions. In order to determine whether our top paths are truly enriched in GWAS regions, we established how many of these paths appear in an equivalent number of random regions that have not been implicated by an autoimmune GWAS. We centered each random region around a SNP that was matched for a similar MAF and LD score as the GWAS tag SNP. We ran this analysis 100 times to define a null distribution for the number of top paths found in a background region. We found that 19 out of 480 top paths was not a significant enrichment (p = 0.44).

**Table 4:** Top causal paths reported in real data analysis that localized within GWAS regions for 8 autoimmune diseases. For each path, we report the chromosome, the RSID of the implicated SNP, the implicated mark type, the posterior probability we assigned to this path, three Z-scores (SNP to mark association, mark to expression association, SNP to expression association), the GENCODE gene around which this region was centered, the ChromHMM [24] annotation for the SNP, and the number of regulatory motifs altered by the SNP, as designated by HaploReg [25].

chr	rsid	GWAS	mark type	posterior	SNP-mark	mark-exp	SNP-exp	gene	chrom state	motifs altered
2	rs2975781	UC, IBD	H3K27ac	1.00	-9.00	5.33	-4.96	GPR35	7Enh	9
8	rs2618481	SLE	H3K27ac	0.94	-6.04	6.59	-3.99	BLK	2TssAFlnk	0
16	rs9927129	Crohn’s, IBD	H3K4me1	0.66	-7.82	-0.79	1.59	RP11-1348G14.2	15Quies	1
6	rs2071889	UC, SLE, MS, RA, IBD	DHS	0.61	6.51	-3.23	-1.78	TAPBP	4Tx	2
16	rs394502	Crohn’s, IBD	H3K4me1	0.44	9.96	-1.59	-2.62	EIF3CL	15Quies	4
1	rs57126490	UC, MS, RA, IBD	DHS	0.43	4.65	-0.14	0.04	PANK4	5TxWk	0
6	rs915654	UC, SLE, Crohn’s, PBC, MS, RA, IBD	H3K4me3	0.42	3.48	5.98	3.51	LTA	7Enh	5
1	rs114312440	Crohn’s	H3K4me3	0.41	-4.54	3.44	-2.79	MTX1	5TxWk	2
3	rs71155551	SLE	H3K27ac	0.39	4.73	3.20	1.27	COPG1	5TxWk	2
1	rs34769708	Crohn’s	H3K4me3	0.39	-4.86	2.13	-2.71	ASH1L	7Enh	3
6	rs13197384	MS	H3K4me3	0.35	6.68	4.44	3.84	AHI1	1TssA	16
6	rs147085011	UC, SLE, PBC, MS, RA, IBD	H3K4me3	0.32	5.11	-0.27	-0.42	RPP21	1TssA	16
16	rs243332	PBC, MS	DHS	0.28	4.45	2.26	0.74	SOCS1	1TssA	9
6	rs575034	RA	H3K4me1	0.23	3.73	3.51	0.85	SLC35B2	1TssA	1
2	rs737231	Crohn’s, Celiac	H3K4me1	0.22	3.59	3.13	2.10	SLC9A4	15Quies	6
5	rs17097187	MS	H3K4me3	0.22	-2.94	6.27	-4.93	PCDHGA1	9Het	4
2	rs737231	IBD	H3K4me1	0.22	3.59	3.13	2.10	SLC9A4	15Quies	6
1	rs2641116	UC, IBD	H3K4me3	0.20	4.57	0.59	1.08	PARK7	4Tx	1
20	rs6115319	MS	H3K27ac	0.11	-5.58	6.39	-4.13	FAM182B	15Quies	0

## Discussion

In this work we proposed a hierarchical fine-mapping framework that integrates three levels of data—genetic, chromatin, and gene expression—to pinpoint SNPs and chromatin marks that may be concordantly influencing gene expression. A key contribution of our approach is the ability to model the correlation structure in the association statistics using a Matrix-variate Normal distribution. Our approach is superior to existing methods, demonstrating the advantage of using a probabilistic approach that takes into account the full sequential model. Moreover, *pathfinder* produces well-calibrated posterior probabilities, and is thus a reliable method for the prioritization of SNPs and marks for functional validation.

We conclude by addressing some of the limitations of our method. Most notably, our method is based upon the SNP→mark→expression assumption. In many genomic regions that show simultaneous evidence for SNP to mark and SNP to gene expression effects, this model will not necessary hold true. In simulations, we show that under the SNP→expression→mark violation, *pathfinder* may identify causal paths very confidently, leading to false positives under the proposed model. When a SNP is in fact independently influencing a mark and gene expression, *pathfinder* is less likely to produce false positives. However, the risk of mis-appropriating our method in this way can be reduced by requiring genomic regions to show evidence for our causal model. We recommend a pre-filtering step before running *pathfinder* on real data that we outline in Methods. In our empirical data analyses, we demonstrate that this two-step regression robustly filters out non-conforming regions. We also acknowledge that, though there are multiple lines of evidence for SNPs influencing expression through local hQTLs, recent works have also emphasized the importance of interactions with distal hQTLs. Thus, developing a systematic way to incorporate data in distal regions with evidence for interactions with a local eQTL would be a fruitful direction. Moreover, *pathfinder* assumes that the true causal SNP and mark within a region are present in the data, which may not always be the case. In this scenario, *pathfinder* will instead place its confidence in the SNP or mark that best correlates with the missing causal SNP or mark in question. Similarly, many epigenetic marks are not themselves causal for gene expression, but are simply correlated to a causal event (e.g., transcription factor binding). It is also often the case that multiple marks at promoter and enhancer regions are concordantly acting to impact gene expression. In these cases, individual marks are not necessarily causal in themselves, but may be viewed as a cause for inter-individual variation or simply correlated to a causal factor. In this light, *pathfinder* aims to identify the epigenetically modifying region so that it can be tested experimentally and/or characterized functionally (for example, to identify the effector transcription factor). We also note that *pathfinder* currently uses an approximation whereby the observed Z-score at the causal SNP is used to estimate the true NCP at the causal SNP (Methods). We leave this to be addressed in future work; this correction will likely further improve the calibration of our method’s credible sets. We note that *pathfinder* only uses individuals for which we simultaneously have genetic, chromatin, and gene expression measurements, thus ignoring eQTL data that has been measured in larger sample sizes. However, eQTL data from larger samples could potentially be used as a prior for expectation of SNP causality or perhaps for validation after running *pathfinder* on real data. Finally, although our analyses showed that H3K4me3 marks are the most informative for fine-mapping, small data set sizes analyzed in this work prohibit us in making definitive conclusions on which mark is most useful leaving such avenues for future work.

## Materials and methods

### Model and likelihood

For each individual, let *h* be the signal value for the causal histone mark and *G* be their vector of genotypes at a region containing *s* SNPs. Let *E* be the individual’s mRNA expression level for the gene at this region and *H* be a vector representing all *t* marks at the region, which contains *h*. Here we analyze all individual peak locations across all available mark types in a joint framework. As such, each of *t* individual marks represents one peak location for a particular mark type. Our causal framework can be modeled as:

$$h=G\beta_{g}+\epsilon_{g}$$ (1)

$$E=H\beta_{h}+\epsilon_{h}$$ (2)

where $\epsilon_{g}∼N\left(0,1-\sigma_{g}^{2}\right)$ and $\epsilon_{h}∼N\left(0,1-\sigma_{h}^{2}\right)$. The vector *β*_*g*_ represents the allelic effects on the causal histone mark whose entries will be non-zero only at the causal SNP. The vector *β*_*h*_ represents the histone mark effects on expression levels whose entries will be non-zero only at the causal histone mark. $\sigma_{g}^{2}$ and $\sigma_{h}^{2}$ represent the variance explained at the SNP-mark and mark-expression levels.

#### Modeling mark to expression associations

We estimate mark to expression effects with linear regression to quantify the strength of association of the *k*th mark through the Wald statistic:

$$Z_{h}^{k}=\frac{\hat{\beta_{h}^{k}}}{SE(\hat{\beta_{h}^{k}})}$$ (3)

$$Z_{h}^{k}∼N\left(\lambda_{h}^{k},1\right)$$ (4)

$$\lambda_{h}^{k}=\frac{\beta_{h}^{k}\sqrt{Var(h^{k})}}{\sigma_{h}}\sqrt{N}$$ (5)

Here, $\hat{\beta_{h}^{k}}$ is the estimated effect size of the causal peak on expression. $\lambda_{h}^{k}$ represents the strength of our signal for causal marks [19]. However, correlations between histone marks will induce a non-zero non-centrality parameters (NCPs) at non-causal histone marks. If we collect all pairwise mark correlations into Σ_*h*_, and let Λ_*h*,*d*_ be the vector of NCPs for all histone marks on expression given causal mark *d*, all summary statistics can be approximated by an MVN.

$$Z_{h}|C_{h}∼N\left(\Sigma_{h}\Lambda_{h,d},\Sigma_{h}\right)$$ (6)

where **C**_**h**_ is an indicator vector containing zeros at all non-causal marks and 1 at the causal mark *d*, and **Σ**_**h**_**Λ**_**h**,**d**_ represents the vector of induced effect sizes at non-causal marks due to inter-mark correlations.

As we do not know the causal effect size **Λ**_**h**,**d**_, we use a normal prior on the causal mark NCPs which can be integrated out as follows:

$$\Lambda_{h,d}|C_{h},\sigma_{h}^{2}∼N(0,\Sigma_{C,h}))$$ (7)

$$\Sigma_{C,h}=\sigma_{h}^{2}\text{Diag}\left(C_{h}\right)+\text{Diag}\left(\epsilon \right)$$ (8)

$$Z_{h}|\Sigma_{h},C_{h}∼(\int N(\Sigma_{h}\Lambda_{h,d},\Sigma_{h})N(0,\Sigma_{C,h})d\Lambda_{h,d})P\left(C_{h}\right)$$ (9)

$$=N\left(0,\Sigma_{h}+\Sigma_{h}\Sigma_{C,h}\Sigma_{h}\right)P\left(C_{h}\right)$$ (10)

Here the prior probabilities of the causal set vector *P*(**C**_**h**_)) is set to be uniform. As a parameter of the model, we set a prior variance explained $\sigma_{h}^{2}$ for the mark effects. We found the method to be fairly robust to variations in this parameter (Fig 5J–5L), and chose a prior variance of 5 for our analyses. In practice, we add an *ϵ* of 0.0001 along the diagonal of Σ_*C*,*h*_ to ensure positive semidefiniteness. Thus, the mark-expression association statistics can be expressed as:

$$Z_{h}|C_{h}∼N\left(0,\Sigma_{h}+\Sigma_{h}\Sigma_{C_{h}}\Sigma_{h}\right)$$ (11)

#### Modeling SNP to mark associations

As before, we estimate SNP to mark effects with linear regression to quantify the strength of association of the *j*th SNP on the *k*th mark through the Wald statistic:

$$Z_{g}^{j,k}=\frac{\hat{\beta_{g}^{j,k}}}{SE(\hat{\beta_{g}^{j,k}})}$$ (12)

$$Z_{g}^{j,k}∼N\left(\lambda_{g}^{j,k},1\right)$$ (13)

$$\lambda_{g}^{j,k}=\frac{\beta_{g}^{j,k}\sqrt{Var(g^{j})}}{\sigma_{g}}\sqrt{N}$$ (14)

Here, $\hat{\beta_{g}^{j,k}}$ is the estimated effect size of the causal SNP on the causal peak. $\lambda_{g}^{j,k}$, the NCP, represents the strength of our signal for causal SNP-mark effects. However, LD between SNPs and correlations between marks will induce non-zero NCPs at non-causal SNP-mark pairs. We collect all pairwise SNP correlations into **Σ**_*g*_ and all pairwise mark correlations into **Σ**_*h*_, and use the Matrix-variate Normal distribution to jointly approximate the association statistics for all SNPs on all marks as:

$$Z_{g}|C_{g},C_{h}∼MN\left(M,\Sigma_{g},\Sigma_{h}\right)$$ (15)

Here, **M** is an *s* × *t* matrix representing association means between all *s* SNPs and all *t* marks, where each entry $M_{j,k}=\Sigma_{g}^{j,c}\Sigma_{h}^{k,d}\lambda_{c,d}$, such that the induced NCP for SNP *j* on mark *k* is just the NCP for causal SNP *c* on causal mark *d*, attenuated by the correlation between SNPs *j* and *c*, as well as the correlation between marks *k* and *d*. Here, rather than integrating out the causal NCPs as we did with the mark-expression associations, we use the observed Z-score for the causal SNP-mark pair to approximate the λ_*j*,*k*_ terms, as the integration is not straightforward in the matrix-variate setting.

#### Computing posterior probabilities for causality

The posterior probability for causality for a given path can be expressed as

$$P\left(C_{h},C_{g}|Z_{g},Z_{h}\right)=\frac{P\left(Z_{g},Z_{h}|C_{h},C_{g}\right)P\left(C_{h},C_{g}\right)}{P(Z_{g},Z_{h})}$$ (16)

A prior can be specified on the probability that a SNP or mark within a fine-mapping region is causal, informed by features like distance to TSS, which is known to correlate with causality [10, 21], or functional annotations. Here we assign this prior to be uniform:

$$P\left(C_{h},C_{g}|Z_{g},Z_{h}\right)=\frac{P(Z_{g},Z_{h}|C_{h},C_{g})}{P(Z_{g},Z_{h})}$$ (17)

$$=\frac{P\left(Z_{h}|C_{h}\right)(P\left(Z_{g}|C_{h},C_{g}\right)}{P(Z_{g},Z_{h})}$$ (18)

We obtain *P*(**Z**_**h**_|**C**_**h**_) from Eq 11 and *P*(**Z**_**g**_|**C**_**h**_, **C**_**g**_) from Eq 15. We then compute *P*(**Z**_**g**_, **Z**_**h**_) by summing over the individual likelihoods for all possible causal paths. Here our method assumes a single causal SNP and mark per region, as we restrict our enumeration to only pairwise causal SNP-mark combinations.

### Simulation framework

We simulated data for 100 individuals over 10,000 50KB regions, using genotypes and LD from 65 YRI individuals obtained through 1000 Genomes [26]. SNP and mark correlations in our simulations were taken from the true correlations exhibited in these regions derived from these individuals. To determine causal status, we randomly chose one SNP and one mark to be causal in each region, thus defining a causal path through the data. Subsequently, we standardized genotypes and simulated values for chromatin marks and gene expression over all 100 individuals.

In order to simulate correlations between histone marks as observed in our empirical data, we drew mark values from an MVN as $N(H_{ind},\epsilon_{g}\Sigma_{h})$, where the means, *H*_*ind*_ = *H*_*c*_Σ_*h*,*c*_, represent the induced values on non-causal marks due to correlations with the causal mark. The mean mark values for the causal mark were generated for each of the 100 individuals as *H*_*c*_ = *β*_*g*_*G*_*c*_, where *G*_*c*_ is the genotype of the individual at the causal SNP, the effect size *β*_*g*_ was drawn from a normal distribution, $N(0,\sigma_{g}^{2})$, with variance set to the desired variance explained by SNPs on marks $\sigma_{g}^{2}=0.25$, with the error term *ϵ*_*g*_ set to $1-\sigma_{g}^{2}$. Finally, the individuals’ values for gene expression are computed as *E* = *β*_*h*_*H*_*c*_ + *ϵ*_*h*_, where *H*_*c*_ is the causal mark value as computed from the MVN, the effect size *β*_*h*_ was set to the desired variance explained from mark to expression $\sigma_{g}^{2}=0.25$, with the remaining error term given by $N(0,1-\sigma_{g}^{2})$.

For simulations in which there were multiple causal SNPs or marks, we randomly drew *m* or *p*, the number of causal SNPs or marks, from a binomial distribution where the expected number of causals per region was set to 1. However, we only included simulations with two or more causals. For multi-causal-SNP simulations, we then randomly selected *m* causal SNPs in the region and simulated chromatin marks and gene expression as described previously, but drew the effect sizes of each SNP as $N(0,\sigma_{g}^{2}/m)$, such that the total expected variance explained remained at 0.25. For multi-causal-mark simulations, we randomly selected *p* causal marks in the region and simulated chromatin marks by defining the means, *H*_*c*_, of each causal mark independently as described for the single-causal simulations. We then computed gene expression by drawing the effect size, *β*_*h*_, of each causal mark from $N(0,\sigma_{g}^{2}/p)$ such that the total expected variance explained remained at 0.25.

### Existing approaches

We benchmark our method against five alternative approaches. Firstly, we compare against the standard overlap analysis whereby hQTLs and eQTLs are independently identified within a region centered around a gene. We follow the protocol outlined in [14]. In this experiment, we computed the best SNP association in each region with every mark measured in the region as well as with the gene expression value for that region. We determined adjusted p-values for each top association by performing permutation tests. We then accounted for multiple testing at the mark level by determining the minimum FDR at which each adjusted p-value would be considered significant. This was estimated via the qvalue package [27]. This procedure resulted in a set of significant SNP-mark associations, as well as one SNP-expression association within the region, as only the top SNP association is retained for each biological phenotype. We then evaluated the number of causal SNPs, marks, and paths that were ultimately included in these candidate sets.

Secondly, we compared against the approach of independently fine-mapping the two levels of data (SNP-mark and mark-expression), and multiplying together pairs of posterior probabilities to produce probabilities of causality for paths. For these independent fine-mapping experiments, we used a simple approach that assumes a single causal variant, approximating posterior probabilities for causality directly from Z-scores [28].

In addition, we compared against a basic ranking approach, where we independently computed SNP-mark, mark-expression, and SNP-expression associations for every SNP and mark within a region. For SNP and mark prioritization, we simply produced a ranking of the SNP-expression and mark-expression posterior probabilities for causality, respectively. For path prioritization, we produced a ranking of the product of SNP-mark and SNP-expression posterior probabilities.

We next compared against a bayesian network model which computes directed association strengths between all possible pairs of nodes in a given network [20]. The method takes as input raw genotype and phenotype values. As nodes, we included all SNPs and marks, as well as the gene expression value, within a region. We allowed only for node pairings directed from SNP to mark or from mark to gene expression. For SNP and mark prioritization, we ranked association strengths over all directed SNP-expression edges and mark-expression edges, respectively. For path prioritization, we produced a ranking of the product of SNP-mark and mark-expression strengths.

Finally, we compared against Coloc, which is designed to identify SNPs that are likely to be causal for multiple traits at once. Specifically, Coloc outputs a posterior probability that a SNP is causal for two arbitrary traits simultaneously. We adapted Coloc for our purposes by running the method on all SNPs independently. For each SNP, the two given traits were (1) gene expression, and (2) a mark value. Thus, we ran Coloc independently for all SNP-mark combinations. This produced a set of posterior probabilities indicating, for each SNP-mark combination, the likelihood that the SNP is causal for both the mark value and gene expression simultaneously. For path prioritization, we ranked these probabilities over all SNP and mark combinations. For SNP and mark prioritization, we marginalized over all marks and SNPs, respectively, producing posterior probabilities for each SNP and mark to be causal independently.

### Real data

The real data analyses were done on 65 YRI individuals whose genotypes were obtained through 1000 Genomes and standardized. PEER-normalized [29] H3K4me1, H3K4me3, H3K27ac, DHS, and RNA expression marks in lymphoblastoid cell lines (LCLs) for these individuals were obtained from [10]. For each gene in the dataset, we computed associations for every SNP-mark, SNP-gene, and mark-gene pair within a 50kb window centered around the gene TSS. On average, each region contained 160 SNPs and 25 marks (across the four mark types—H3K4me1, H3K4me3, H3K27ac, and DHS—whose peak values we analyzed together in each region). Overall, from 14,669 50kb regions, we filtered for regions that exhibited evidence for our sequential model where SNPs affect chromatin marks, which in turn affect gene expression. Specifically, for each region we performed a two-stage regression where we first regressed gene expression on all chromatin marks, and (2) regressed the proportion of expression explained by the chromatin marks on each SNP. If at least one SNP had a low p-value for association (*p* < 0.05/*n*.*snps*) to the proportion of gene expression explained by chromatin data, we kept this region for our real data analysis. After this filtering procedure, we retained 1,317 regions.

We obtained motif annotations from HaploReg [25] and ChromHMM annotations from the NIH Roadmap Epigenomics Consortium [30]. When comparing annotations of top prioritized paths with those of random paths, we established corresponding background paths by choosing a random SNP/mark combination at every region where a top path was reported.

For GWAS analyses, we explored regions whose tag SNP was associated to an autoimmune trait with *p* < 5 × 10^−8^. Associations were obtained from recent literature for eight autoimmune phenotypes [31–36]. For each of *pathfinder*’s top reported paths, we determined whether the corresponding SNP was contained within any of the GWAS regions in our dataset. In order establish a null distribution for this statistic, we ran the same analysis for random regions in the genome not overlapping with the GWAS regions in our dataset. Specifically, for each GWAS region, we randomly selected a SNP in the same chromosome matched for MAF (*ϵ* = 0.01) and LD score (*ϵ* = 0.001) with the GWAS tag SNP. We established a window around this matched SNP corresponding to the window size of the GWAS region. Finally, we determined the number of top paths that fell within these random regions. We repeated this experiment 100 times to establish the null distribution of this measurement and calculated a p-value using a Z-test.

## Supporting information

S1 Table 50%, 90%, and 99% credible sets for SNP-, mark-, and path-mapping in simulations. We compare *pathfinder* to basic eQTL mapping with respect to the size of their credible sets, averaged across all regions. Standard errors are included next to each measurement.
(TIF)

S2 Table 50%, 90%, and 99% credible sets for SNP-, mark-, and path-mapping for simulations using YRI LD and CEU LD. We compare *pathfinder’s* performance on simulations using SNP LD from YRI versus from CEU, with respect to the size of its credible sets, averaged across all regions. Standard errors are included next to each measurement.
(TIF)

S3 Table Aggregate probability mass assigned to DHS, H3K4me1, H3K4me3, and H3K27ac. We compare the total probability amassed at all peaks for each mark type after running *pathfinder* on empirical data. We display both the raw probability mass and the average mass contribution per peak location for each mark type.
(TIF)

S4 Table 50%, 90%, and 99% credible sets for SNP-, mark-, and path-mapping for real data analysis in CEU individuals. We compare *pathfinder’s* performance on PEER-corrected data and raw data, with respect to the size of its credible sets, averaged across all regions. Standard errors are included next to each measurement.
(TIF)

S1 Fig 50%, 90%, and 99% credible sets for SNP-, mark-, and path-mapping, in comparison to independent fine-mapping. We compare *pathfinder* to the technique of independently fine-mapping the two levels of data, with respect to the calibration of their credible sets (A, C, E) and the size of their credible sets (B, D, F).
(TIF)

S2 Fig Performance of the method in the presence of two anti-correlated mark effects. We assess *pathfinder*’s behavior in simulations with respect to SNP-, mark-, and path-mapping (A-C) when an additional peak in the region has an effect on expression that is opposite from the mediating peak in question, compared with regions in which the effect of the additional peak has a matching sign.
(TIF)

S3 Fig Comparison of ranking approaches in response to violations of the causal model. We compare *pathfinder*’s response to violations of the causal model against the behavior of other ranking approaches. Causal models are illustrated to the left of the figure. (A, D, G, J, M, P, S) display SNP-mapping accuracy. (B, E, H, K, N, Q, T) display mark-mapping accuracy. (C, F, I, L, O, R, U) display path-mapping accuracy.
(TIF)

S4 Fig Runtime analysis. *pathfinder*’s runtimes on empirical data with respect to the number of SNPs, marks, and paths within a region (A-C). We plot each simulation as a point and fit a line to all points.
(TIF)

S5 FigObserved $h_{g}^{2}$ in empirical data.We report the distribution of SNP-mark (A), mark-expression (B), and SNP-expression (C) $h_{g}^{2}$ levels observed across all top paths selected by *pathfinder*.(TIF)

S6 Fig Association plots for top region reported by *pathfinder* in real data, spanning a 50kb region centered around the NDUFA12 TSS. (A) Mark-expression Z-scores are reported for all marks. (B) SNP-mark Z-scores are reported for the top mark chosen by *pathfinder*. The implicated SNP, rs835044, lies 6bp downstream of the NDUFA12 TSS.
(TIF)

S7 Fig Spatial relationships between SNP, mark, and TSS in top paths reported by *pathfinder* vs random paths, stratified by mark type. (A-C) DHS. (D-F) H3K4me1. (G-I) H3K4me3. (J-L) H3K27ac.
(TIF)
