# Supplementary figures and images for: Methods for fine-mapping with chromatin and expression data
Source: PLoS Genet. 2018 Feb 26;14(2):e1007240. doi: 10.1371/journal.pgen.1007240 (PMC5843356; doi:10.1371/journal.pgen.1007240)

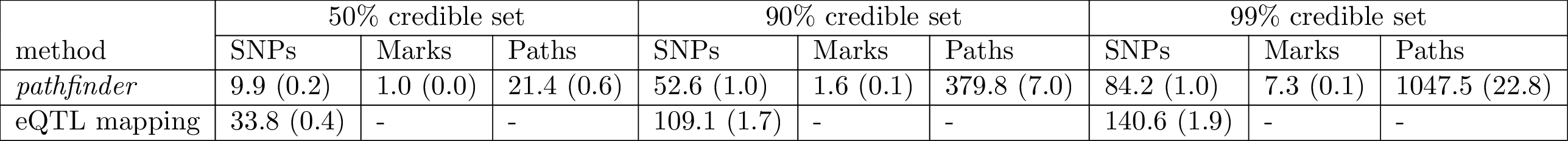

Supplement: S1 Table — We compare pathfinder to basic eQTL mapping with respect to the size of their credible sets, averaged across all regions. Standard errors are included next to each measurement. (TIF) [file pgen.1007240.s001.tif]

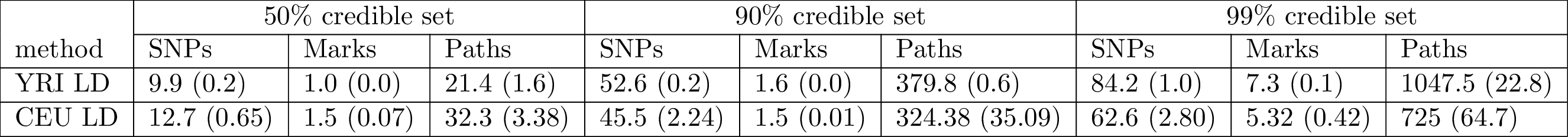

Supplement: S2 Table — We compare pathfinder’s performance on simulations using SNP LD from YRI versus from CEU, with respect to the size of its credible sets, averaged across all regions. Standard errors are included next to each measurement. (TIF) [file pgen.1007240.s002.tif]

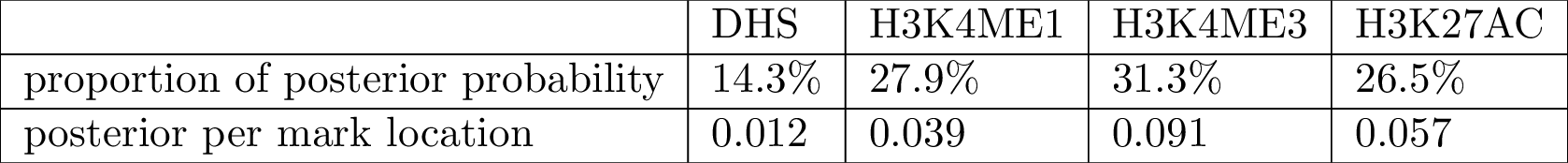

Supplement: S3 Table — We compare the total probability amassed at all peaks for each mark type after running pathfinder on empirical data. We display both the raw probability mass and the average mass contribution per peak location for each mark type. (TIF) [file pgen.1007240.s003.tif]

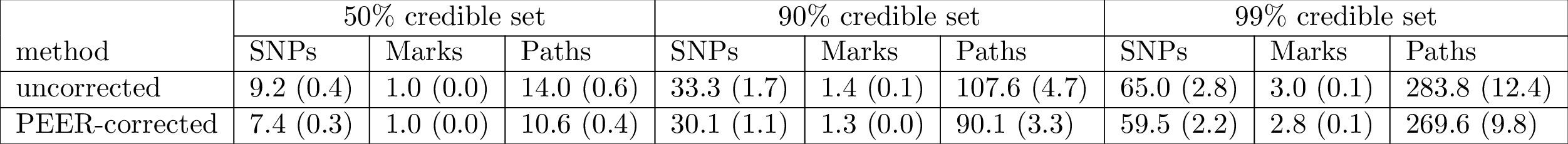

Supplement: S4 Table — We compare pathfinder’s performance on PEER-corrected data and raw data, with respect to the size of its credible sets, averaged across all regions. Standard errors are included next to each measurement. (TIF) [file pgen.1007240.s004.tif]

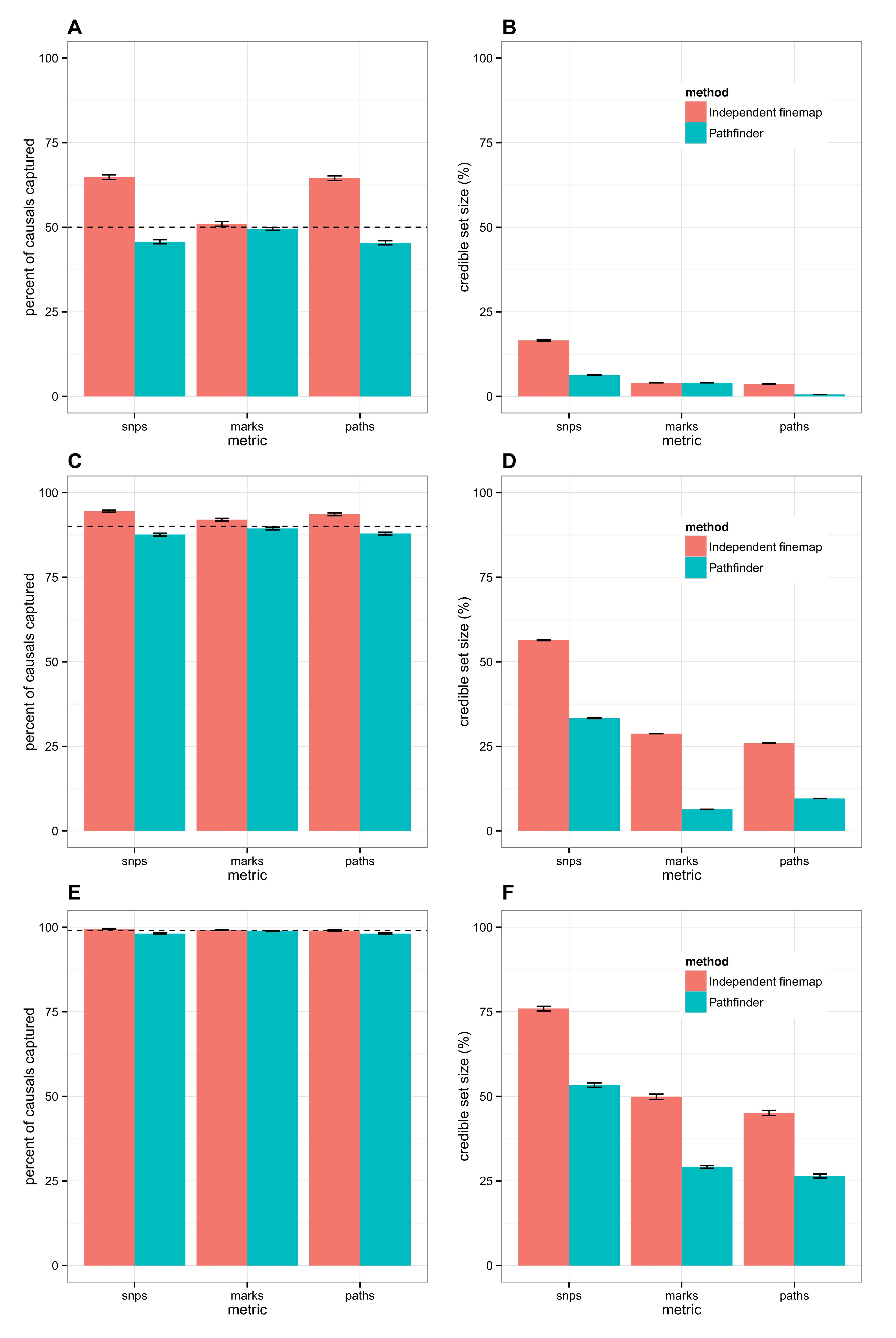

Supplement: S1 Fig — We compare pathfinder to the technique of independently fine-mapping the two levels of data, with respect to the calibration of their credible sets (A, C, E) and the size of their credible sets (B, D, F). (TIF) [file pgen.1007240.s005.tif]

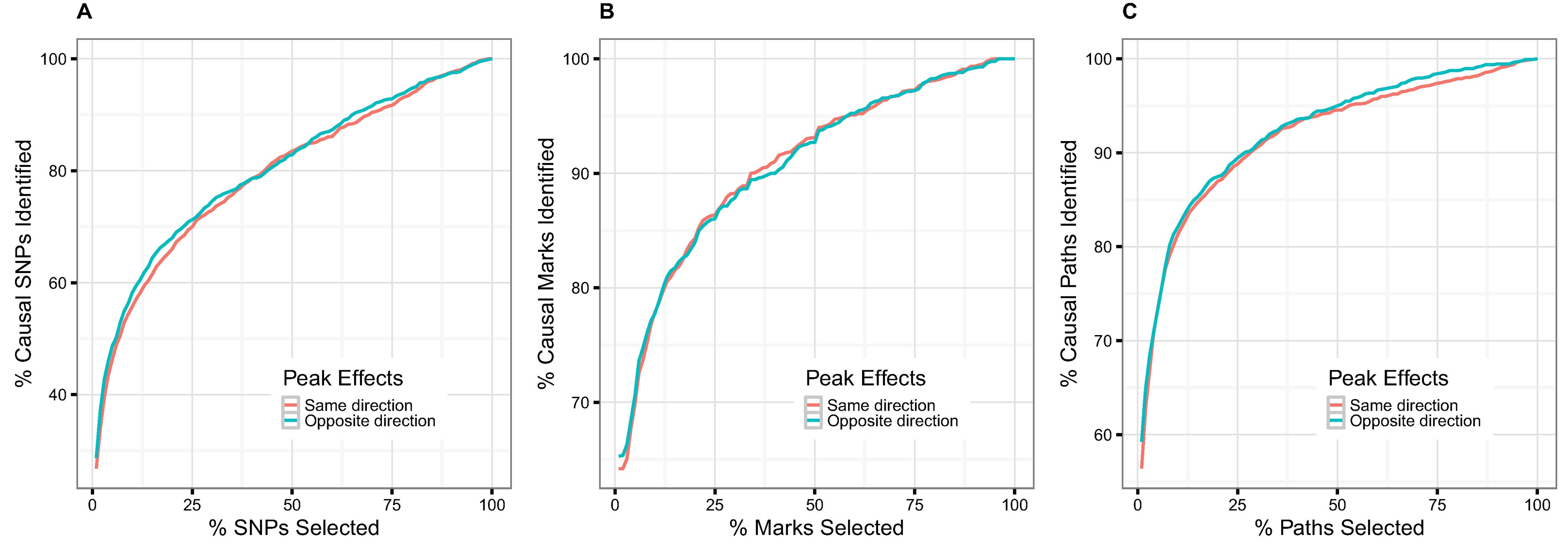

Supplement: S2 Fig — We assess pathfinder’s behavior in simulations with respect to SNP-, mark-, and path-mapping (A-C) when an additional peak in the region has an effect on expression that is opposite from the mediating peak in question, compared with regions in which the effect of the additional peak has a matching sign. (TIF) [file pgen.1007240.s006.tif]

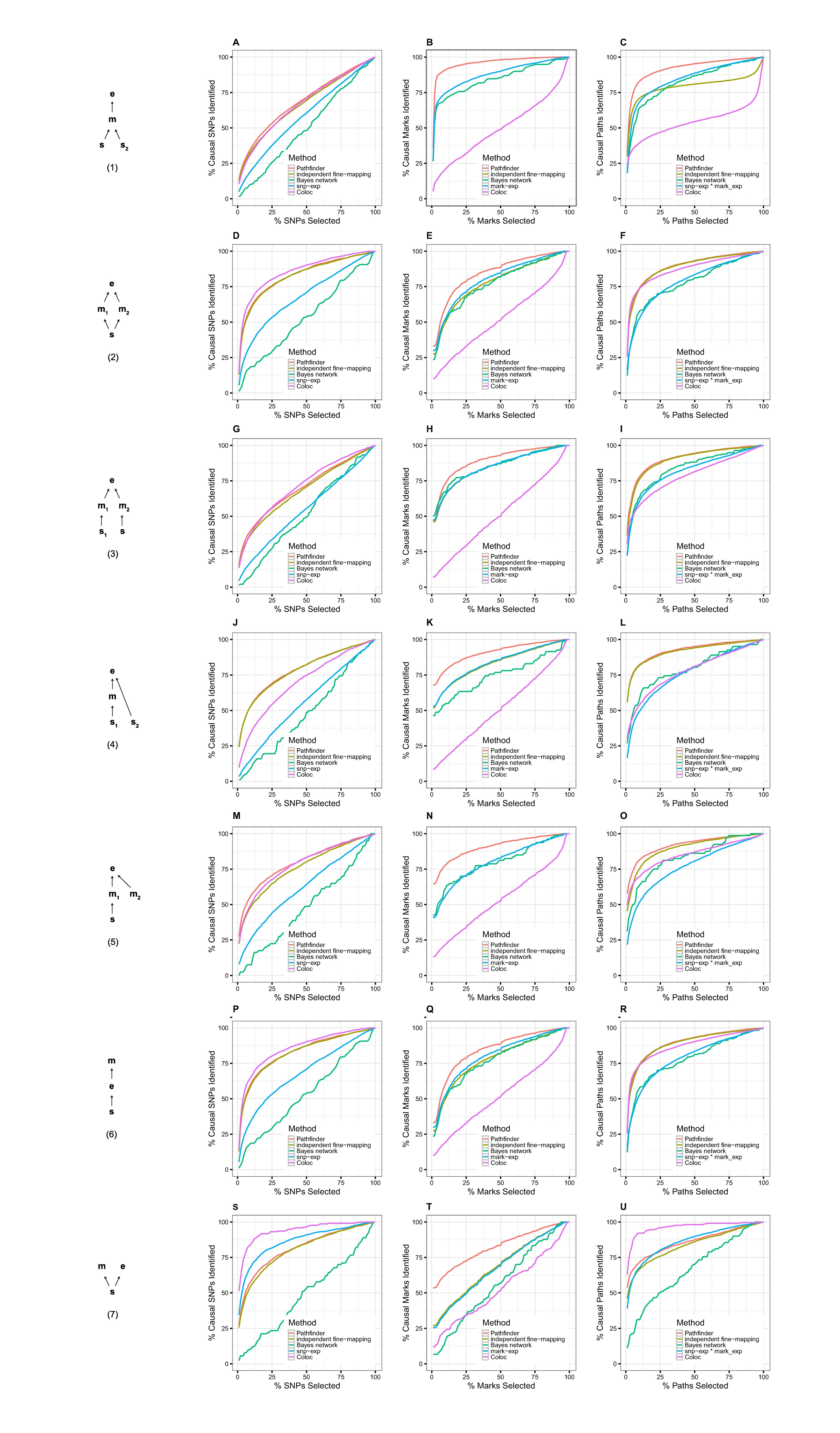

Supplement: S3 Fig — We compare pathfinder’s response to violations of the causal model against the behavior of other ranking approaches. Causal models are illustrated to the left of the figure. (A, D, G, J, M, P, S) display SNP-mapping accuracy. (B, E, H, K, N, Q, T) display mark-mapping accuracy. (C, F, I, L, O, R, U) display path-mapping accuracy. (TIF) [file pgen.1007240.s007.tif]

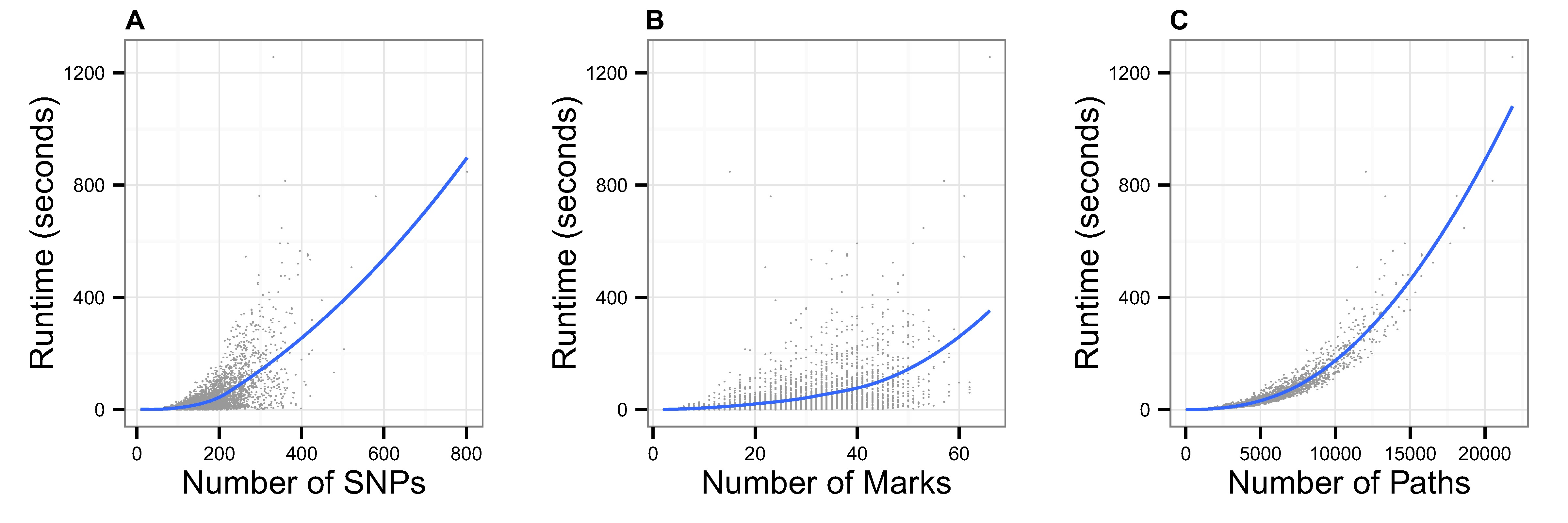

Supplement: S4 Fig — pathfinder’s runtimes on empirical data with respect to the number of SNPs, marks, and paths within a region (A-C). We plot each simulation as a point and fit a line to all points. (TIF) [file pgen.1007240.s008.tif]

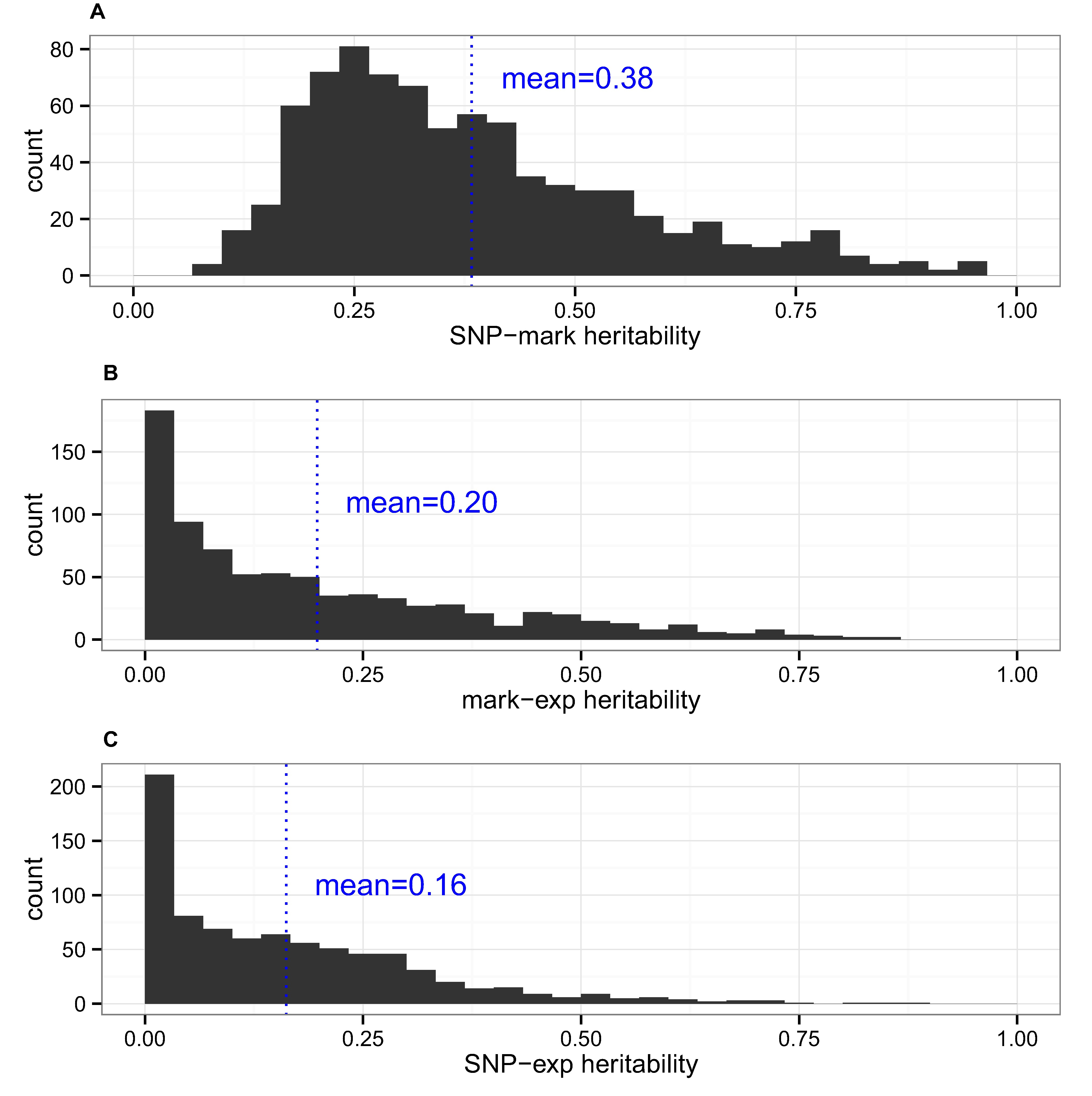

Supplement: S5 Fig — We report the distribution of SNP-mark (A), mark-expression (B), and SNP-expression (C) hg2 levels observed across all top paths selected by pathfinder. (TIF) [file pgen.1007240.s009.tif]

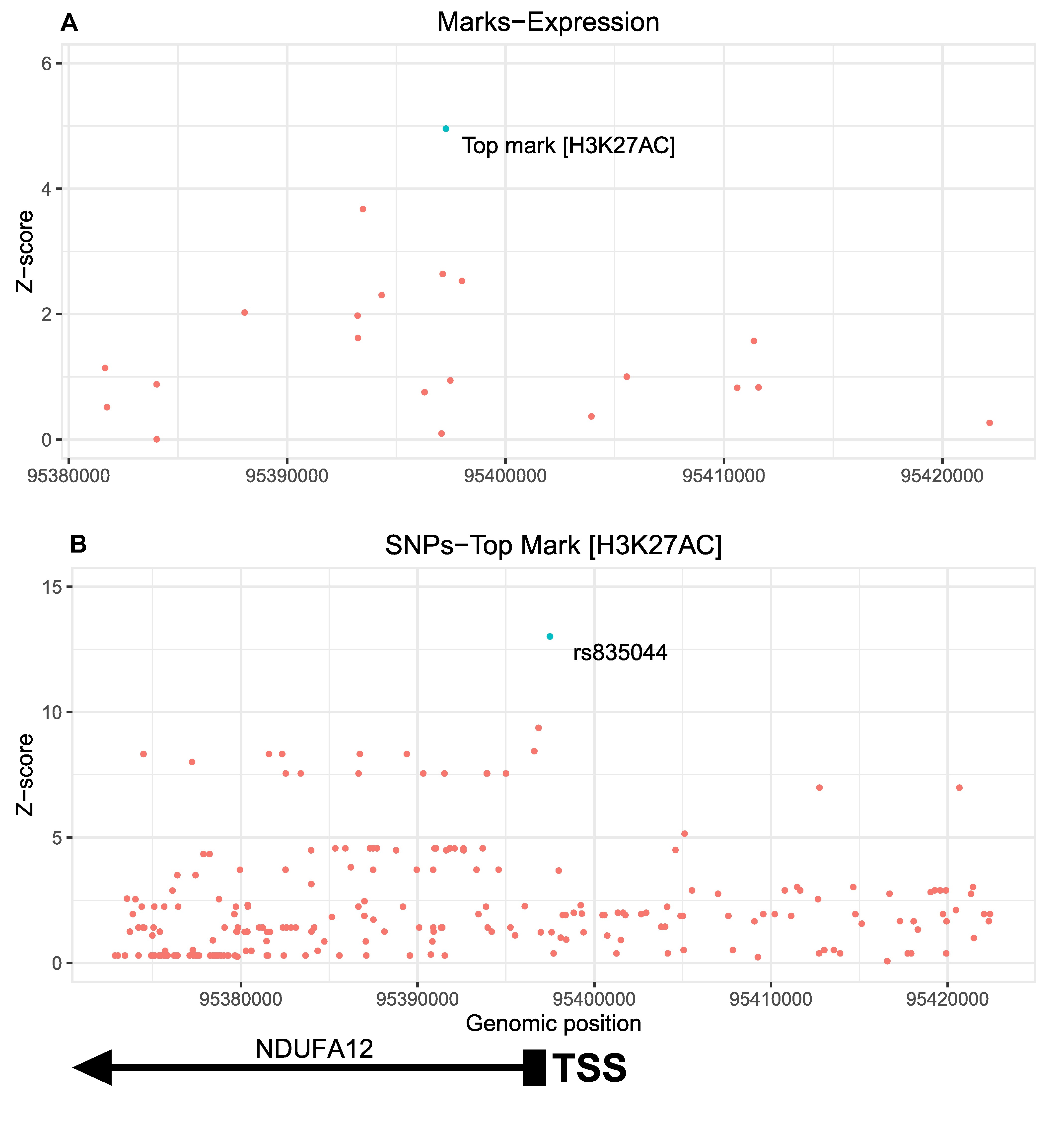

Supplement: S6 Fig — (A) Mark-expression Z-scores are reported for all marks. (B) SNP-mark Z-scores are reported for the top mark chosen by pathfinder. The implicated SNP, rs835044, lies 6bp downstream of the NDUFA12 TSS. (TIF) [file pgen.1007240.s010.tif]

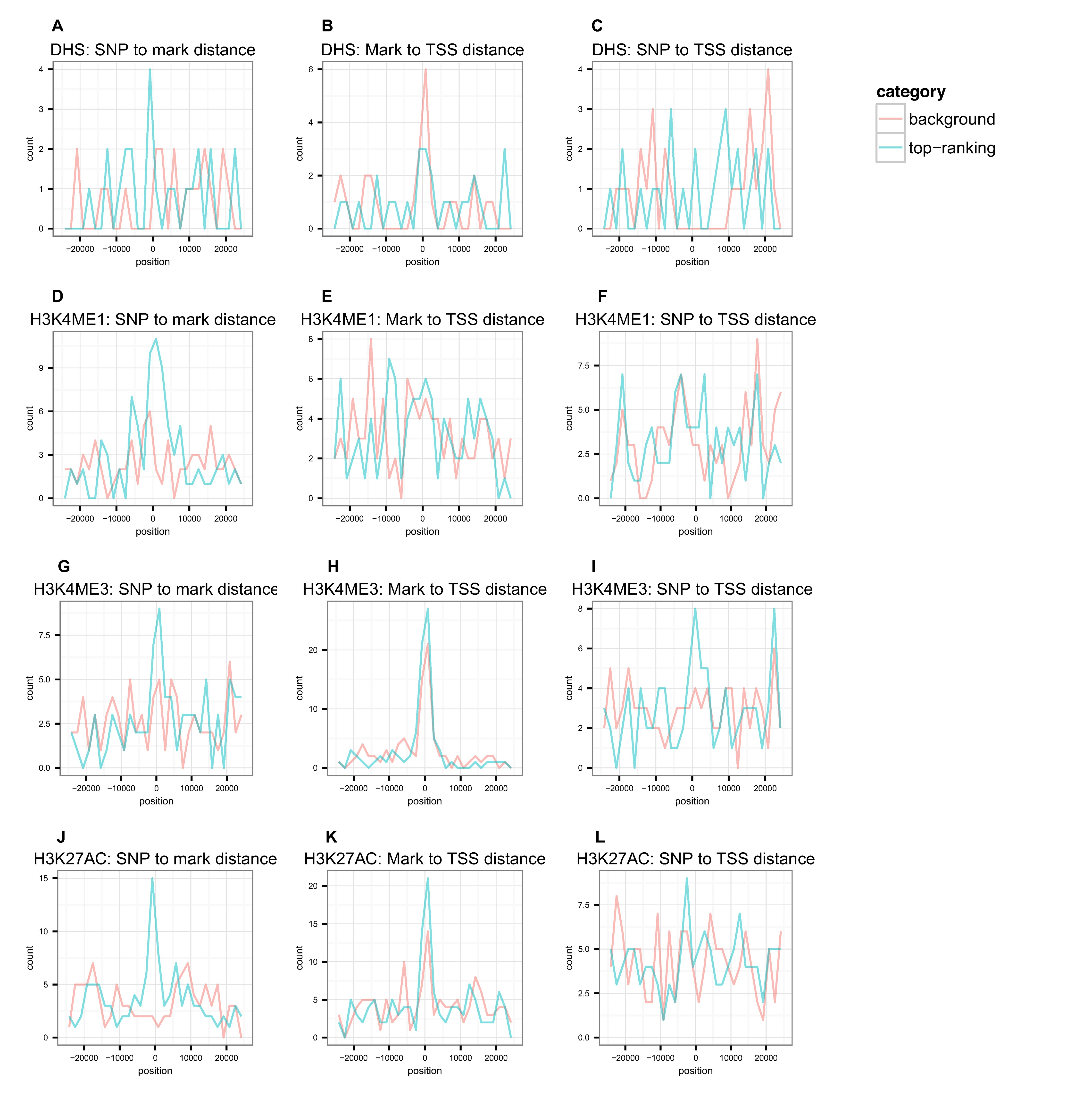

Supplement: S7 Fig — (A-C) DHS. (D-F) H3K4me1. (G-I) H3K4me3. (J-L) H3K27ac. (TIF) [file pgen.1007240.s011.tif]
